# The Role of the Gut Microbiome in Clinical Outcomes of Colorectal Cancer: A Systematic Review (2020–2025)

**DOI:** 10.32604/or.2025.070281

**Published:** 2026-02-24

**Authors:** Iara Santos, Joana Liberal, Paulo Teixeira, Diana Martins, Fernando Mendes

**Affiliations:** 1 Coimbra Health School (ESTeSC), Polytechnic University of Coimbra, Coimbra, 3046-854, Portugal; 2 H&TRC—Health & Technology Research Center, Coimbra Health School, Polytechnic University of Coimbra, Coimbra, 3045-043, Portugal; 3 Pathology Department, Centro Hospitalar e Universitário de Coimbra, Coimbra, 3000-075, Portugal; 4 Faculty of Medicine, University of Coimbra, Coimbra, 3004-535, Portugal; 5 Coimbra Institute for Clinical and Biomedical Research (iCBR) Area of Environment Genetics and Oncobiology (CIMAGO), Biophysics Institute of Faculty of Medicine, University of Coimbra, Coimbra, 3045-043, Portugal; 6 Center for Innovative Biomedicine and Biotechnology, University of Coimbra, Coimbra, 3000-548, Portugal; 7 European Association for Professions in Biomedical Sciences, Brussels, 1000, Belgium

**Keywords:** Colorectal neoplasms, gastrointestinal microbiome, host microbial interactions, drug therapy, systematic review

## Abstract

**Background:**

The Colorectal Cancer (CRC) pathogenesis and therapeutic efficacy are influenced by the gut microbiome, making it a promising biomarker for predicting treatment responses and adverse effects. This systematic review aims to outline the gut microbiome composition in individuals with CRC undergoing the same therapeutic regimen and evaluate interindividual microbiome profile variations to better understand how these differences may influence therapeutic outcomes.

**Methods:**

Key studies investigating the microbiome’s role in therapeutic approaches for CRC were searched in both PubMed and Cochrane databases on 12 and 22 March 2025, respectively. Eligible studies included free full-text English-language randomized clinical trials and human observational studies reporting on gut microbiome composition and treatment outcomes. RoB 2 and ROBINS-I were employed in the evaluation of bias for randomized trials and observational studies, respectively. Data extracted was narratively analyzed.

**Results:**

Six studies involving a total of 361 individuals were included. Therapeutic interventions, either standard treatments and/or those targeting the gut microbiome, generally increased probiotic taxa and reduced pro-carcinogenic bacteria. However, no consistent pattern of improved clinical outcomes was observed, suggesting that treatment mechanisms, the tumor’s nature, and individual characteristics play critical roles in microbiome modulation.

**Conclusion:**

The gut microbiome holds significant potential in clinical settings. Nonetheless, further research is needed to better understand its functional aspects and to consider the influence of treatment mechanisms, the tumor’s nature, and individual characteristics as modulators, in order to optimize clinical outcomes.

## 1 Introduction

Colorectal cancer (CRC) is among the most prevalent cancers worldwide and the second leading cause of cancer-related mortality [1], with a significant morbidity and recurrence rate associated [2,3]. Modifiable and non-modifiable factors influence CRC risk. While age and genetics play a role, the rising incidence in younger adults suggests a greater importance of modifiable contributors [4], particularly gut microbiome alterations [1]. The gut microbiota includes 10^14^ microorganisms, which primarily reside in the gastrointestinal tract [5]. Beyond the microbiota, the gut microbiome encompasses its structural elements, genes, and metabolites [6]. It symbiotically associates with the host and is crucial in metabolism, immune regulation, and behavioral responses [7,8].

Treatment strategies for CRC are tailored to the individual patient’s disease stage, tumor location, and presence of metastasis. In early-stage CRC, tumor resection is the first-line therapy. In contrast, stages II, III, and IV often necessitate the addition of systemic therapy to the treatment regimen, such as chemotherapy, immunotherapy, and radiotherapy [2,9].

A bidirectional relationship exists between the gut microbiome and these treatments. The gut microbiota, known to play a role in CRC pathogenesis [10–13], can also play a critical role in modulating the efficacy and toxicity of various cancer therapies. Conversely, it is increasingly recognized that cancer treatments themselves can alter the composition and activity of the gut microbiota [8,14], with tumor microenvironment and surgical procedures promoting a state of dysbiosis characterized by reduced microbial diversity and richness, which can significantly impair therapeutic efficacy and exacerbate toxicity [7,14,15].

This dysbiotic state has significant clinical consequences. In surgical patients, emerging evidence suggests a link between postoperative complications and gut microbiome alterations [16,17]. Preoperative interventions, like bowel preparation and antibiotic prophylaxis, disrupt microbial composition [18,19]. Given the microbiome’s role in inflammation and tissue repair [20], such dysbiosis may contribute to negative outcomes [21], which in turn represents a risk factor of reduced overall survival and increased risk of recurrence [22]. For systemic treatments, the mechanisms by which the microbiome influences treatment outcomes are varied. Dysbiosis facilitates microbial translocation across the compromised intestinal barrier, potentially triggering inflammation [23]. Additionally, direct microbial metabolism and its byproducts can alter therapeutic pharmacokinetics, efficacy, and toxicity by modulating metabolic pathways, reactivating inactive metabolites, and producing toxic products [8,24,25]. Furthermore, the microbiome modulates both innate and adaptive immune responses, influencing the efficacy of immunotherapy by either enhancing antitumor activity or contributing to therapeutic resistance [12,14,15,26].

The gut microbiome may represent a promising prognostic biomarker and therapeutic target, with its modulation offering potential to enhance efficiency and reduce adverse effects on CRC treatment. Therefore, this review aims to outline the gut microbiome composition in individuals with CRC undergoing the same therapy regimen, compare interindividual variations, and understand how these differences may influence therapeutic outcome.

## 2 Materials and Methods

### 2.1 Literature Research Strategy

To perform this systematic review, we used the Preferred Reporting Items for Systematic Reviews and Meta-Analyses (PRISMA) Statement [27], and the completed checklist is provided as Supplementary Material. This systematic review protocol was registered at the International Platform of Registered Systematic Review and Meta-analysis Protocols (INPLASY) with the digital object identifier (DOI) number 10.37766/inplasy2025.7.0042 on 10^th^ July 2025. Even though this represents a retrospective registration, no major deviations occurred.

The formulation of the scientific question that guides this research project is structured using the PICO model [28]. The PICO framework comprises “P” for population, “I” for intervention, “C” for comparison, and “O” for outcomes. The population comprised patients with colorectal cancer. The intervention was defined as the characterization of gut microbiome composition in individuals undergoing the same therapeutic regimen. The comparison focused on interindividual variations in microbiome profiles, while the outcome was to evaluate how these differences may influence clinical outcomes. In this way, primary outcomes included clinical endpoints, such as response to therapy and treatment-related toxicity. Secondary outcomes included gut microbiome diversity indices, taxonomic composition, microbial metabolites, and other biomarkers.

A literature search was conducted using PubMed and Cochrane databases on 12 and 22 March 2025, respectively, with the following keywords: “Gastrointestinal Microbiome” (D000069196), “Colorectal Neoplasms” (D015179), “Host Microbial Interactions” (D000076662), “Drug Therapy” (D004358), “Immunotherapy” (D007167) and “Radiotherapy” (D011878). Table 1 outlines the research methodologies employed, which involved using Medical Subject Headings terms and combinations of keywords to obtain the relevant literature for this review. The literature search conducted on PubMed also included the filters “Free Full-Text” and “English”, as well as the time range between “January 2020” and “January 2025”.

**Table 1:** The strategy used to obtain the literature used in this review by combining the keywords (Medical Subject Headings terms) “Gastrointestinal Microbiome”, “Colorectal Neoplasms”, “Host Microbial Interactions”, “Drug Therapy”, “Immunotherapy” and “Radiotherapy”, and use the Boolean operators

Medical subject headings terms	Boolean operators		
	And	Or	Not
**Colorectal neoplasms**	Gastrointestinal microbiome, Drug therapy		Immunotherapy, Radiotherapy
**Colorectal neoplasms**	Gastrointestinal microbiome, Immunotherapy		Drug therapy, Radiotherapy
**Colorectal neoplasms**	Gastrointestinal microbiome, Radiotherapy		Immunotherapy, Drug therapy
**Colorectal neoplasms**	Host microbial interactions, Drug therapy, Cancer vaccines		Immunotherapy, Radiotherapy
**Colorectal neoplasms**	Host microbial interactions, Immunotherapy		Drug therapy, Radiotherapy
**Colorectal neoplasms**	Host microbial interactions, Radiotherapy		Immunotherapy, Drug therapy

Note: MeSH Terms, Medical Subject Headings Terms.

The restriction of literary research to only PubMed and Cochrane databases was deliberate, as both provide extensive coverage of peer-reviewed studies relevant to the research question. Although expanding the search to additional databases could have offered further advantages, the decision was guided by time and resource constraints. Within these limits, PubMed and Cochrane were considered sufficient to capture the majority of pertinent literature.

### 2.2 Selection Criteria and Review Methods

The screening of the previously identified articles was conducted on the “PICO Portal literature review platform” [29], which enhances the efficiency of the review process by consolidating all articles and their corresponding assessments in a centralized platform.

Studies were eligible to be included in the review based on specific inclusion and exclusion criteria designed to ensure that only articles providing pertinent information were included. Free full-text articles published in English between January 2020 and January 2025 were eligible for inclusion.

The search was limited to English-language publications, as English represents the primary language of international scientific communication and ensures accessibility of findings to the wider research community. In addition, only free full-text articles were considered, reflecting resource constraints during the review process. Although these criteria may exclude other relevant studies, this approach facilitated feasibility and transparency. The restricted search to studies published within the last five years was based on the rapid expansion of microbiome research in oncology, with substantial methodological advances and scientific discoveries. This ensured that the included studies reflected current scientific standards, clinically relevant outcomes, and up-to-date therapeutic approaches.

**Figure 1:**
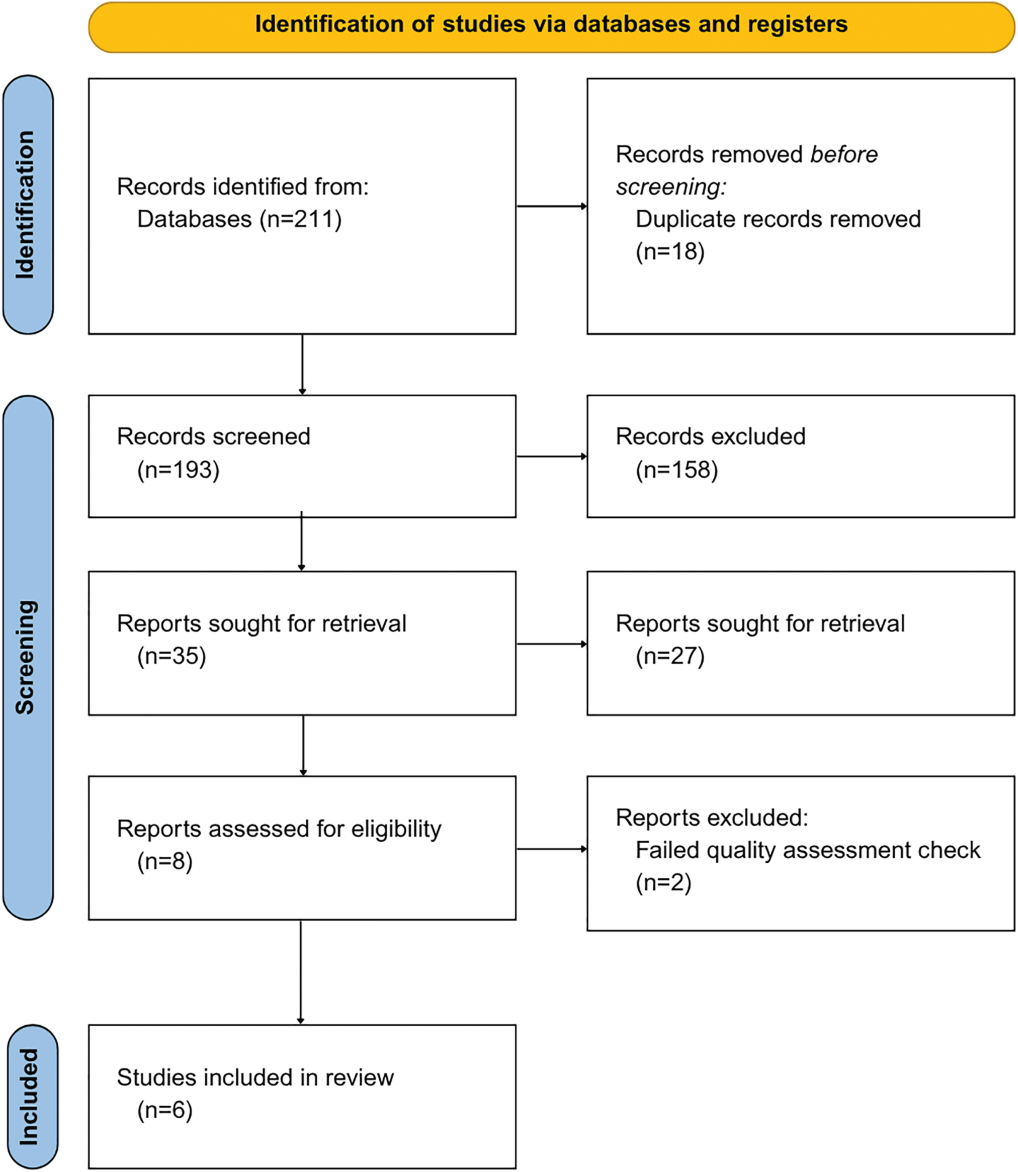
PRISMA systematic review, including the identification of pertinent literature, the number of articles screened, and the total number of articles excluded and included in the review

Concurrently, randomized controlled trials (RCTs) and human observational studies were included. Simultaneously, any literature that didn’t meet these criteria was excluded. Furthermore, studies were excluded if they were considered to have limitations such as a high risk of bias, incomplete data reporting, or an unclear study objective, resulting in six studies included in this systematic review as described in Fig. 1.

Given the anticipated heterogeneity of study designs, interventions, and outcome measures, a quantitative meta-analysis was not feasible. The studies varied in the interventions assessed, sequencing methodologies applied, and outcomes reported, and the small number of available studies further limited the potential for statistical pooling. For these reasons, we prespecified a narrative synthesis approach. This approach enabled the identification of patterns specific to each therapeutic strategy and highlighted differences in microbiome composition, diversity, and associated clinical outcomes, while ensuring transparent and clinically coherent interpretation.

### 2.3 Quality Assessment Review

The risk of bias assessment for the studies included in this systematic review was conducted using the Risk-of-Bias Visualization (Robvis) tool [30], a comprehensive and widely used tool to assess the quality and risk of bias in research studies.

**Figure 2:**
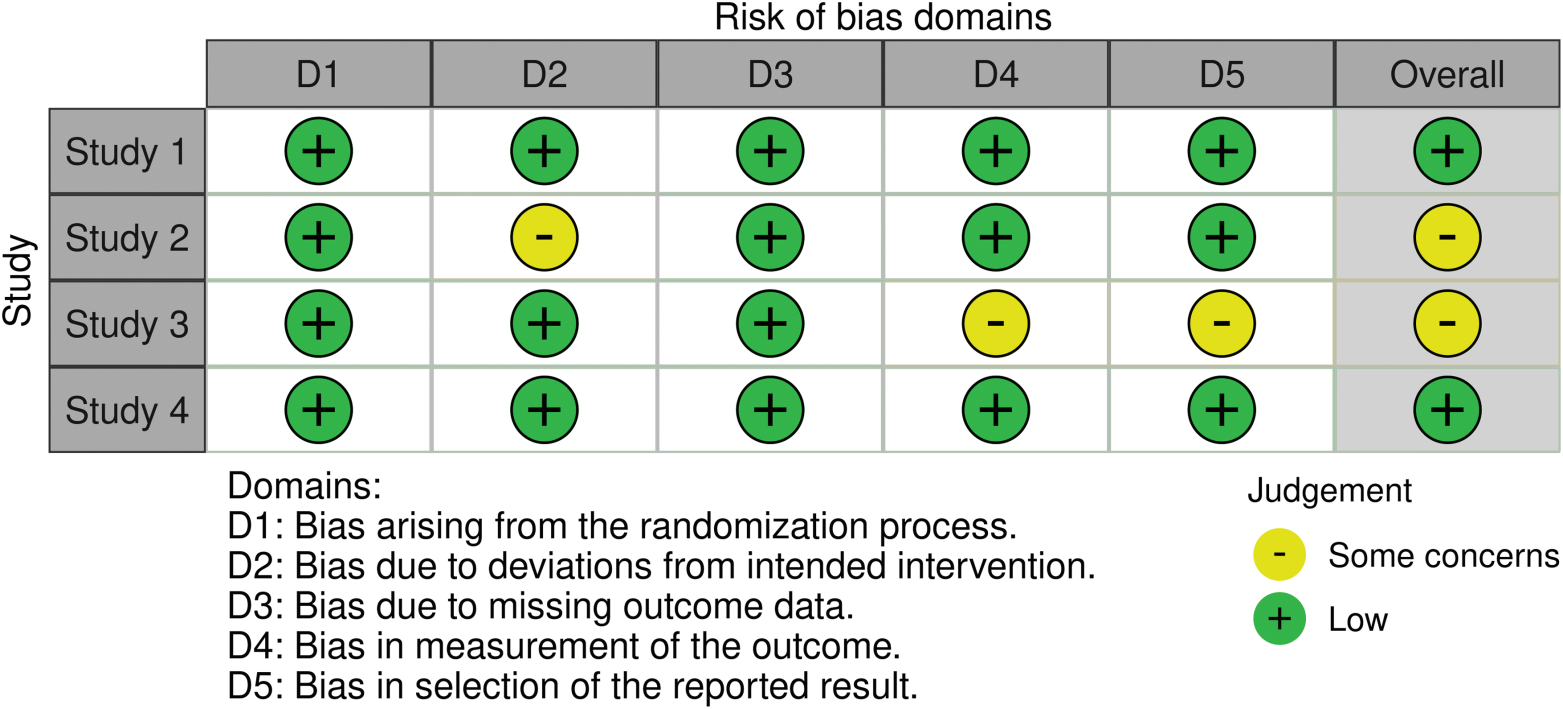
Traffic light plot from randomized controlled trials, using the RoB 2 tool

**Figure 3:**
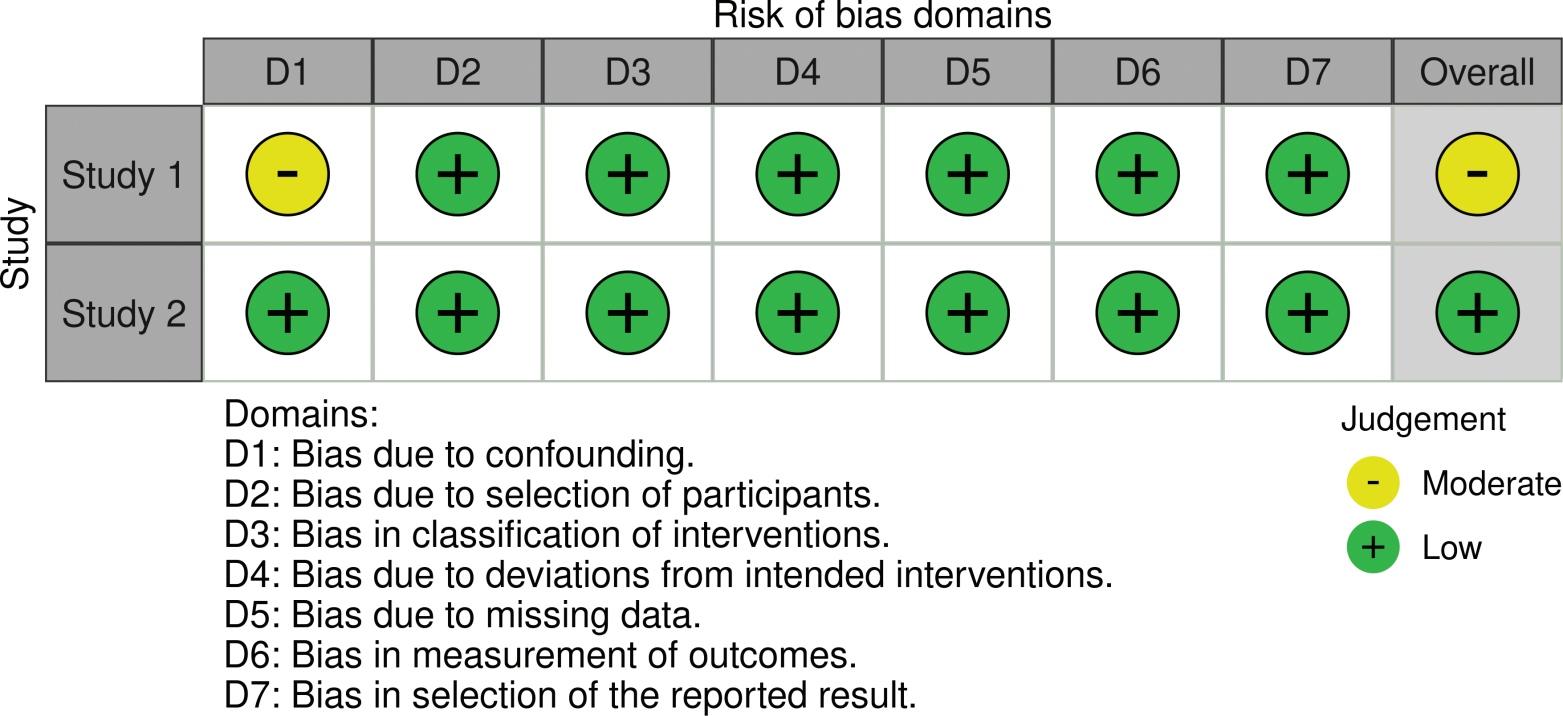
Traffic light plot from human observational studies, using the ROBINS-I tool

As both RCT and human observational studies were included in this review, different Robvis tools were used to provide a clear and concise overview of the risk of bias assessment of the included studies. Both traffic light plots generated by Robvis are represented in Figs. 2 and 3, providing a clear and concise overview of the risk of bias assessment of the included studies.

The RoB 2 tool [31] offers a structured framework for evaluating risk of bias in randomized trials, encompassing five domains where bias may be introduced. This tool was applied to assess bias in the RCT studies included (Fig. 2).

The ROBINS-I tool [32] was employed in the evaluation of bias in non-randomized studies. It evaluates seven domains where bias may be introduced. It was used to assess bias in the included human observational studies, as shown in Fig. 3.

To evaluate the certainty of evidence compiled in this review it was applied the Grading of Recommendations, Assessment, Development and Evaluations (GRADE) approach [33]. This framework considers five domains for downgrading the certainty rating: risk of bias, inconsistency, indirectness, imprecision, and publication bias, across the body of evidence for each outcome. While observational studies were included, and these traditionally commence at a low level of certainty, the certainty may begin at an initial high level when the studies are rigorously evaluated for risk of bias using an appropriate tool, such as the ROBINS-I [34]. Certainty of evidence was classified as high, moderate, low, or very low, and a Summary of Findings (SoF) table was produced to present outcome-specific ratings, which can be found at the end of the Results section.

Regarding the assessment of publication bias, according to current guidelines (Cochrane Handbook for Systematic Reviews of Interventions) [35] tests for funnel plot asymmetry are not recommended when fewer than 10 studies are included in the synthesis, as the power of the tests is too low to distinguish chance from real asymmetry. Adhering to these guidelines, and noting the absence of obvious selective reporting, no publication bias was detected among the included studies.

## 3 Results

The gut microbiome intervenes in multiple stages of CRC pathogenesis. Understanding the potential of the intestinal microbiome to enhance treatment efficacy and reduce adverse effects in CRC may be a promising direction for optimizing cancer therapy outcomes.

This review presents the main findings from six studies. For each treatment modality, we first provide a brief overview, followed by a synthesis of the reported results. When relevant, additional literature is referenced to contextualize and deepen the interpretation of the findings. Table 2 summarizes the methodological characteristics of all included studies.

**Table 2:** The table presents an overview of the studies included in this review. The information compiled includes study design, number of participants, tumor staging, therapy, sampling, sequencing, end points, and main findings

Study	Study design	N	Stage	Intervention	Sampling	Sequencing	End points	Main findings
Žukauskaitė et al., 2024 [36]	RCT	N = 40 OP (n = 18) RE (n = 20)	Not reported	The OP group ingested 4 L of Macrogol 4000 (73.69 mg/L) starting the afternoon before surgery. The RE group was given 2 L of 0.9% sodium chloride. Both groups received antibiotic prophylaxis 30–60 min before surgery (2 g of Cefazolin and 500 mg of Metronidazole).	Fecal samples were collected one day before (baseline), and on postoperative days 6 (POD6) and 30 (POD30) to assess gut microbiota composition.	V1–V2 regions of 16S rRNA sequencing	Postoperative morbidity, α- and β-diversity	Treatment-induced transient dysbiosis in both groups. Postoperative morbidity is independent of MBP. *E. faecalis* is linked to infections.
Aarnoutse et al., 2022 [37]	Human observational study	N = 33	Stage IV	Three cycles of Capecitabine (1000–1250 mg/m^2^, twice daily on days 1–14 in a 3-week cycle) with or without Bevacizumab (7.5 mg/kg on day 1 every 3 weeks).	Fecal samples were collected one or two days before the start of the capecitabine cycle (T1), between days 7–14 of the third cycle (T2), and at day 20 or 21 of the third cycle (T3).	V4 region of 16S rRNA sequencing	Tumor response, α- and β-diversity, microbiota composition, toxicity	High interindividual heterogeneity but no consistent microbiome pattern with response. NR with higher grades of fatigue compared to responders.
Sánchez-Alcoholado et al., 2021 [38]	Human Observational Study	N = 60 Healthy Controls (n = 20) CRC Patients (n = 40)	Stages II–III	Five weeks of Neoadjuvant Radiochemotherapy with Pelvic Radiation Therapy (50 Gy in fractions of 2 Gy/session) and Capecitabine (825 mg/m^2^/12 h), followed by surgical resection.	Fecal and blood samples were collected at baseline (T0), two and four weeks after starting RCT treatment (T1 and T2), and seven weeks after treatment’s ending (T3).	Multiple variable regions (V2, 3, 4, 6–7, 8, and 9) of 16S rRNA sequencing	Tumor regression grade, α- and β-diversity, microbiota diversity and composition, SCFA, polyamines and their acetyl derivatives, zonulin	R and NR showed different intestinal microbial compositions, corresponding to different metabolic functions and producing SCFA.
Huang et al., 2023 [39]	RCT	N = 100 Placebo (n = 50) Intervention (n = 50)	Stages I–III	Both groups underwent surgical resection followed by the first cycle of adjuvant XELOX Intervention group took one capsule of probiotic (0.5 g and contained over 0.5 × 10^6^ CFU of *B. infants*, *L.acidophilus*, *E. faecalis*, and over 0.5 × 10^5^ CFU of *B. cereus*) 3 times per day from the third postoperative day to the end of the first chemotherapy course, while Placebo group took placebo tablets.	Fecal samples were collected pre-surgically from fifty randomized subjects and from all participants at study completion. Blood samples were taken before surgery, on the first postoperative day, and before and after the first chemotherapy course.	V4 region of 16S rRNA sequencing	α- and β-diversity, microbiota composition, SCFA, gastrointestinal complications	Probiotics preserved diversity and counteract treatment-induced dysbiosis, seem to contribute to increased SCFA levels and reduced treatment-related GI complications, and don’t affect chemotherapy efficacy.
Li et al., 2020 [40]	RCT	N = 70 Control (n = 37) Intervention (n = 33)	Stages I–III	The Control group received routine treatment followed by surgery. The Intervention group receives routine treatment and 250 mL (twice daily) of GQD (*Radix Pueraria*e (15 g), *Scutellariae Radix* (9 g), *Coptidis Rhizoma* (9 g), and *Liquorice* (6 g)) for 7 days, followed by surgery.	Fecal samples were collected before and after the intervention in the treatment group. Blood samples were collected at the beginning of the study in the control group, and before and after GQD administration in the treatment group. Surgical specimens were used.	V4 region of 16S rRNA sequencing	α- and β-diversity, microbiota composition, immune markers, inflammation markers, intestinal barrier proteins, subjective and abdominal symptoms	Intervention implicates a microbial shift, which reflects functional differences in the GQD enhances immunity, reduces inflammation, enhances intestinal barrier function and improves symptoms.
Bellerba et al., 2022 [41]	RCT	N = 60 Placebo (n = 32) Intervention (n = 28)	Stages I–III	Supplementation of Vitamin D_3_ 2000 IU or placebo once daily for one year.	Blood and fecal samples were collected simultaneously at baseline and after twelve months (M12).	Shotgun metagenomic sequencing	25(OH)D levels, gut microbiome composition, DFS	Vitamin D supplementation increased 25(OH)D levels, altered gut microbial composition and function, and interacted with host factors (age, sex, BMI). Baseline *F. nucleatum* correlated with poorer DFS.

Note: BMI, Body Mass Index; DFS, Disease Free-Survival; GQD, Gegen Qinlian Decoction; GI, Gastrointestinal; Gy, Gray; MBP, Mechanical Bowel Preparation; NR, Non-Responders; OP, Osmotic diarrhea-inducing Oral Preparation; PODs, Postoperative Days; R, Responders; RCT, Randomized Clinical Trial; RE, Rectal Enema; SCFA, Short-Chain Fatty Acids. Underlined text indicates the specific components of each therapeutic regimen.

### 3.1 Surgical Intervention and Mechanical Bowel Preparation

Surgical resection is a standard procedure for all CRC stages; tumor resection is the first-line therapy. The Žukauskaitė et al. [36] conducted a pilot two-arm randomized clinical trial designed to compare oral preparation (OP) and rectal enema (RE) impact on the gut microbiota and their potential association with postoperative complications in patients undergoing surgery for left-sided CRC patients [36]. All patients received mechanical bowel preparation (MBP), being randomly allocated to either Osmotic diarrhea-inducing Oral Preparation (OP) group (4 L oral macrogol 4000; n = 20) or Rectal Enema (RE) group (2 L 0.9% sodium chloride; n = 20), followed by standard antibiotic prophylaxis (2 g of Cefazolin and 500 mg of Metronidazole) 30 to 60 min preoperatively. Fecal samples were collected one day before (baseline), and on postoperative days 6 (POD6) and 30 (POD30) [36].

Žukauskaitė et al. [36] showed that both MBP and colorectal resection have a significant impact on the microbiome’s composition of the microbiome in a 30-day time frame. Moreover, it was shown that MBP (independently of whether it was OP or RE) led to significant within-group compositional shifts from baseline to POD6 and from POD6 to POD30, suggesting that the treatment induced transient but notable microbiota alterations [36]. In the OP group, there was a transient decrease in *Dialister* and an increase in *Citrobacter* genus, both of which returned to baseline levels by POD30. Persistent genus-level shifts included an increase of *Collinsella* and decreased *Porphyromona*s, with increased *Eubacterium coprostanoligenes* and *Eubacterium hallii* across both postoperative time points. However, the reliability of the long-term observations at POD30 is limited by deviations from intended interventions, as only 12 participants in this group were analyzed at this time point, representing a 40% decrease rate from the original cohort. In the RE group, POD6 increases were seen in *Actinomyces*, *Enterococcus*, *Parabacteroides*, and *Ruminococcus 2* genera, with most reverting to baseline by POD30, except *Ruminococcus 2*, which continued to increase [36]. However, the mechanism by which they intervene is poorly understood.

The bulk of evidence supports that MBP can cause widespread and potentially lasting compositional changes [42,43]. In a study conducted in patients undergoing colonoscopy, it was noted that MBP led to significant changes in the gut microbiome composition. While most of these changes reverted to baseline at the 1-month mark, there was also a persistent alteration in a reduction in some bacterial families [44]. It is noteworthy that the study highlights the possibility that the gut microbiome alterations could result from the MBP, surgical intervention, or a combination of both [36]. Surgical prophylaxis with cefazolin is relatively common and used to target most gram-positive coccis and some gram-negative, while metronidazole mainly targets anaerobic bacteria, specifically *B. fragilis*. In another RCT, MBP with prophylactic antibiotic administration was associated with greater shifts in gut microbiome composition and reduced overall postoperative complications compared with exclusive MBP [45].

Currently, these gut microbiome alterations have been linked to postoperative complications [16,17]. Žukauskaitė et al. [36] reported similar postoperative morbidity between groups: 39% (n = 15) of patients developed complications, 32% (n = 12) developed infections, and 8% (n = 3) experienced postoperative ileus. In patients with postoperative infections (regardless of MBP type), higher relative abundance of the *Actinomyces* genus, *Sutterella* uncultured species, and *Enterococcus faecalis (E. faecalis)* species was found on POD6. Additionally, *E. faecalis* levels increased significantly on POD6 and returned to baseline by POD30 in both groups, with a steeper increase in the OP group [36]. This goes in line with what is reported in literature, and highlights the important role of MBP, antibiotic administration and surgical resection in long-term microbiome compositional alterations.

### 3.2 Chemotherapy and Radiochemotherapy

While studies conducted by Aarnoutse et al. [37] and Sánchez-Alcoholado et al. [38] are designed to evaluate a therapeutic regimen on CRC patients, without microbiome-directed interventions, Huang et al. [39] introduces a probiotic intervention. However, in all of them, the treatment regimen includes capecitabine.

Capecitabine is an antimetabolite directed to epithelial cells, working as an analogous of natural pyrimidine and consequently blocking DNA and RNA synthesis in cancer cells, which leads to decreased proliferation of those [46]. Since its metabolization occurs preferentially in tumor tissue, high and effective intratumorally fluorouracil (5-FU) concentrations are reached, without high systemic exposure to 5-FU [47]. It is important to note that capecitabine is a DNA-damaging agent and thus affects all rapidly dividing cells, leading to significant toxicity and limiting its duration of use [48]. Multiple cellular and molecular changes have been reported to play crucial roles in the lack of CRC response to capecitabine treatment, one of them being the tumor microenvironment, which includes the gut microbiome [49]. Furthermore, CRC cells inevitably develop resistance to chemotherapy agents, at which point additional lines of therapy are needed [48].

Aarnoutse et al. [37] conducted a prospective study on metastatic CRC patients to evaluate the chemotherapeutic capecitabine impact on response, toxicity and gut microbiota diversity and composition. In the study, patients underwent chemotherapy treatment with or without Bevacizumab [37]. This is in line with the international guidelines, which recommend capecitabine with or without bevacizumab as first-line treatment for patients with metastatic colorectal cancer for whom more intensive treatment is not appropriate [50]. Bevacizumab is a humanized monoclonal antibody against Vascular endothelial growth factor (VEGF); therefore, it blocks tumor-mediated angiogenesis by limiting the tumor’s blood supply [48]. An RCT associated with bevacizumab inclusion in the treatment, in detriment of only the XELOX regimen, not only with better efficacy of the treatment but also modulation of intestinal microbiome composition, increased gastrointestinal motility of patients, and reduced oxidative stress and adverse events [51].

In Aarnoutse et al. study, tumor response was assessed before and at the end of three cycles of capecitabine through Computed Tomography (CT) or Magnetic Resonance Imaging (MRI) by means of Response Evaluation Criteria in Solid Tumors (RECIST), while gut microbiota constitution was monitored at three distinct times: before the first cycle (T1), between day 7–14 of the third cycle (T2), and at day 20 or 21 of the third cycle (T3). Results showed a Partial Response (PR) in 18% (n = 6), Stable Disease (SD) in 76% (n = 25) and Progressive Disease (PD) in 3% (n = 1). Consequently, six patients were classified as responders (R) and twenty-six as non-responders (NR).

In this context, Aarnoutse et al. reported that α-diversity did not differ significantly between Responders and Non-Responders before and during treatment. Furthermore, no significant changes over three cycles of capecitabine were observed [37].

Considerable heterogeneity was observed in individuals’ gut microbiota structure; however, there was no significant association between treatment response and overall microbiota structure on phylum or genus levels. These findings should be interpreted cautiously, as the analysis did not fully adjust for important confounding variables such as sex, age, prior antibiotic exposure, or number of metastases. Despite significant inter-individual heterogeneity, capecitabine treatment did not induce consistent shifts in microbiota composition. Furthermore, although intra-individual microbiota alterations were observed throughout treatment, those were not consistent and appeared to be influenced by external factors [37]. This last finding will be discussed later in the Discussion section.

Treatment-related adverse effects (AE) such as peripheral sensory neuropathy, hand-foot syndrome, oral mucositis, and bone marrow toxicity increased significantly over the study period. Karnofsky performance score was significantly lower after three cycles of capecitabine. Between Responders and Non-Responders, this last group indicated higher fatigue grades than responders [37].

The Sánchez-Alcoholado et al. [38] conducted a prospective study on CRC patients to identify the possible relationship between the gut microbiome, the fecal Short-Chain Fatty Acids (SCFA) levels, the serum levels of the polyamines and their acetyl derivatives, and the intestinal permeability to neoadjuvant radiochemotherapy (nRCT) outcome. This therapy combines radiotherapy, targeting cancer cells by inducing DNA damage, with chemotherapy. While healthy cells can also be affected, they typically possess better repair mechanisms than neoplastic cells. For stage II/III rectal cancer, current evidence supports nRCT as superior to adjuvant therapy [52], with capecitabine being a widely used radiosensitizer [53].

In this study, all CRC patients completed the 5-week nRCT, which included Pelvic Radiation Therapy (50 Gray (Gy)) in fractions of 2 Gy/session) and oral Capecitabine (825 mg/m^2^/12 h) and underwent surgical resection. Fecal and blood samples were collected simultaneously at four distinct times: at baseline (T0), two and four weeks after starting RCT treatment (T1 and T2, respectively), and seven weeks after treatment’s ending (T3) [38]. Tumor response after nRCT was then determined in surgical samples, which subclassified individuals according to Tumor Regression Grades (TGR): 70% (n = 28) were classified as Responders (TGR 1–2) and 30% (n = 12) as Non-Responders (TGR 3–4). It is worth noting that this study included fecal samples from twenty healthy patients that matched the CRC patients’ characteristics [38].

At baseline, healthy controls had higher gut microbiota diversity and richness than CRC patients. Additionally, analysis of β-diversity showed that the two cohorts had significantly different genus compositions of gut bacteria [38]. At T0, CRC displayed at the genus level a significantly higher relative abundance of *Prevotella*, *Fusobacterium*, and *Escherichia*, while *Bacteroides*, *Roseburia*, *Ruminococcus*, *Faecalibacterium*, *Bifidobacterium*, and *Blautia* were significantly decreased. At the species level, CRC patients showed elevated levels of *Fusobacterium nucleatum (F. nucleatum)*, *Bacteroides fragilis (B. fragilis)*, and *Escherichia coli (E. coli)*, whereas *Bifidobacterium bifidum (B. bifidum)* and *Faecalibacterium prausnitzii* (*F. prausnitzii*) were significantly reduced [38]. Between baseline and different time-points, α-diversity didn’t show significant differences in the levels of richness and diversity, as neither β-diversity showed significant differences in the gut microbial community.

However, when compared between the Responders and Non-Responders groups, the Responder group exhibited significantly higher microbial diversity and richness at the genus level after treatment. Additionally, β-diversity analysis demonstrated a significant difference in the genus-level composition of intestinal microbiota between the Responders and Non-Responders groups [38].

At the genus level, the Responders group showed an increase in *Ruminococcus*, *Bifidobacterium*, *Roseburia*, and *Faecalibacterium*, while the Non-Responders group showed increased *Prevotella*, *Fusobacterium*, *Escherichia*, *Bacteroides*, and *Klebsiella* [38]. At the species level, the Responders group exhibited higher abundance of *B. bifidum*, *Ruminococcus albus (R. albus)*, and *F. prausnitzii*. In contrast, the Non-Responders group showed higher levels of *Prevotella copri*, *E. coli*, *F. nucleatum*, and *B. fragilis* [38].

Kyoto Encyclopedia of Genes and Genomes (KEGG) pathway enrichment analysis revealed distinct overrepresented biological pathways in each group. The Responders group showed enrichment of genes related to energy metabolism, carbohydrate metabolism, xenobiotic biodegradation and metabolism pathways, and membrane transport. On the other hand, the Non-Responders group had enrichment of genes related to lipid metabolism, amino acid metabolism pathways, metabolism of cofactors and vitamins, folate biosynthesis, glycan biosynthesis and metabolism, lipopolysaccharide biosynthesis proteins, cellular processes and signaling that contain cell motility and secretion, oxidative phosphorylation, and pathways of cancer [38]. Significant differences in the serum levels of several polyamines and their acetyl derivatives, fecal levels of SCFA, and zonulin levels were found in both the Responders and Non-Responders patients post-treatment. At the metabolite level, there were significant changes in the levels of N1-acetylspermidine (N1-AcSPD) and spermine in both the Responders and Non-Responders within-group, while the serum levels of N8-acetylspermidine (N8-AcSPD) only varied in the Non-Responders group. When compared, the Non-Responders patients had a significant increase in the levels of spermine, N1-acetylspermine (N1-AcSP), N1, N12-diacetylspermine (N1, N12-DiAcSP), N1-AcSPD, N1, N8-diacetylspermidine (N1, N8-DiAcSPD), and N1-acetylputrescine (N1-AcPUT) [38]. Analysis of the fecal levels of SCFA revealed significant differences in the concentrations of acetic, butyric, isobutyric, valeric, isovaleric, and hexanoic acids between the Responders and Non-Responders study groups at post-treatment. Within-group comparison, Responders had increased fecal concentrations of acetic and butyric acid, while serum zonulin levels only increased in the Non-Responders group [38].

Emerging research suggests that the gut microbiome composition may influence response to nRCT, with higher levels of butyrate-producing bacteria observed in responders [54]. However, the understanding of the role of the gut microbiome in chemoradiotherapy responses remains nebulous.

In Sánchez-Alcoholado et al. study, when a correlation analysis was performed, *F. prausnitzii* and *R. albus* were positively correlated to fecal levels of butyrate in the Responders group. Findings from patient-derived organoids indicated that butyrate may enhance radiotherapy efficacy while protecting healthy mucosa, potentially reducing treatment-related toxicity [55], which is consistent with the findings.

On the other hand, *F. prausnitzii* was negatively correlated to the serum levels of spermine and N1, N12-DiAcSP [38]. In the Non-Responders group, *B. fragilis* was positively correlated with the concentration of propionic acid. *B. fragilis* and *F. nucleatum* were also positively correlated with N1, N12-DiAcSP, and N8-AcSPD levels. Furthermore, increased zonulin levels were correlated with *Prevotella copri* [38].

Upon building a predictive model based on the overall gut microbiota profile using the species-level abundance data. *F. nucleatum and B. fragilis* were overrepresented in Non-Responders patients, while *R. albus, B. bifidum*, and *F. prausnitzii* were biomarkers of Responders patients [38].

### 3.3 Microbiome-Targeted Interventions: Probiotics and Herbal Medicine

Huang et al. [39] and Li et al. [40] studies, distinct from the previous two, are designed to evaluate the modelling potential of certain interventions in the intestinal microbiome and, indirectly, mitigate gut microbiome alterations induced by CRC and associated therapeutic regimen.

Huang et al. [39] conducted a randomized, single-blind, placebo-controlled prospective study to evaluate whether a probiotic combination could mitigate chemotherapy-associated gastrointestinal complications and gut microbiota dysbiosis in CRC patients. All participants underwent surgical resection followed by capecitabine and oxaliplatin (XELOX) chemotherapy. Oxaliplatin is a potent inducer of immunogenic cell death [56] and enhances the efficacy of 5-FU [57]; RCT have demonstrated improved survival with oxaliplatin-5-FU combinations compared to 5-FU alone [58]. Administered postoperatively, adjuvant chemotherapy targets residual micro-metastases to reduce recurrence risk [59].

The intervention group (n = 50) received probiotic capsules three times daily from postoperative day 3 through the end of the first chemotherapy cycle, while the control group (n = 50) received placebo tablets. Probiotic capsules comprise *B. infantis, L. acidophilus, E. faecalis* (0.5 × 10^6^ CFU), and *B. cereus* (0.5 × 10^5^ CFU). Probiotics are live microbial supplements intended to confer health benefits to the host by improving intestinal microbial balance [21]. This is done either directly, via metabolic products, or indirectly, through immunomodulation. Although most probiotic strains do not permanently colonize the gut, they are known to modulate immune responses, restore intestinal barrier function, and reduce intestinal inflammation [21,60]. In this way, probiotic supplementation directly modulates the composition of the gut microbiome by introducing bacteria tended as beneficial to shift its composition towards a healthier state.

The duration of intervention was about six weeks, including two weeks of chemotherapy. Fecal samples were collected from fifty randomized subjects before surgical resection and from all participants at the end of the study. Infection status within six weeks of intervention and patient gastrointestinal symptoms during the two-week chemotherapeutic period were recorded [39].

The gut microbiome appears to influence oxaliplatin efficacy. Studies have shown reduced antitumor effects in antibiotic-treated mice, while butyrate supplementation can enhance CD8^+^ T cell-mediated immunity and improve chemotherapy responses [61]. On the other hand, Huang and colleagues found no significant differences in routine blood indices between groups, indicating that probiotics did not alter chemotherapy efficacy [39].

In terms of α-diversity, the placebo group showed reduced microbial richness post-treatment, while the probiotic group maintained stable richness and diversity. Between-group comparisons showed higher richness and diversity in the probiotic group at study completion. β-diversity analysis suggested some separation between pre-treatment and post-treatment samples, unrelated to intervention, although there were no clear distinctions for the fecal microbial communities among all groups [39].

Before treatment, CRC patients displayed a higher abundance of *Roseburia*, *Phascolarctobacterium*, and *Lactobacillus* [39]. After radical surgery and chemotherapy, treatment regimen led to reduced *Prevotella*, *Lactobacillus*, and *Roseburia*, and increased *Akkermansia* and *Lachnospiraceae_Clostridium* in Placebo group. In contrast, the probiotic group showed restoration of these depleted genera and significant increases in *Bifidobacterium, Streptococcus*, and *Blautia*. When comparing Placebo group and Probio group, it is clear that Placebo group was enriched in *Ruminococcus* and *Enterococcus*, while Probio group had decreased *Faecalibacterium*, *Fusobacterium* and *Sutterella*. These results indicate that probiotic supplementation helped counteract gut microbiota disruption caused by surgery and chemotherapy [39]. The dominant fecal SCFA (acetic, propionic, and butyric acids) were significantly reduced by XELOX chemotherapy but were dramatically elevated in the probiotic group compared to placebo. In the probiotic group, correlation analysis showed positive associations between SCFA levels and the genera *Phascolarctobacterium, Lactobacillus*, and *Roseburia* and negative associations with *Akkermansia* and *Sutterella* [39].

In literature, chemotherapy-induced diarrhea has been linked to decreased α-diversity and microbial richness [62]. Reflecting this, Huang and colleagues identified treatment-related adverse effects, including diarrhea, being increased in the placebo group, which presented lower α-diversity after the treatment [39].

Li et al. [40] conducted a case control study performed on CRC patients to assess the effects of Gegen Qinlian decoction (GQD) on immune function, inflammation and intestinal barrier function. Seventy patients were randomly assigned to a control group (n = 37) or a treatment group (n = 33). While both groups received routine treatment and elective surgery upon admission, the treatment group was also administered oral GQD (250 mL, twice daily) for 7 days before surgery. GQD comprises four medicinal herbs: *Radix Puerariae* (15 g), *Scutellariae Radix* (9 g), *Coptidis Rhizoma* (9 g), and *Liquorice* (6 g). Blood samples were collected at the beginning of the study in the control group, and before and after GQD administration in the treatment group. Surgical specimens were used to assess intestinal barrier function. Fecal samples were collected only in the treatment group, and before and after the intervention. Subjective and abdominal symptoms were monitored throughout the intervention [40]. Although the effectiveness of herbalism is still nebulous, in laboratory settings, it has been shown to have anti-inflammatory and antimicrobial effects [63].

In this study, α-diversity and β-diversity were significantly reduced in the post-treatment group. At the genus level, enrichment of *Bacteroides, Akkermansia*, and *Prevotella* and a reduction in *Megamonas* and *Veillonella* were observed. KEGG pathway analysis indicated alterations in pathways related to energy metabolism, immunity, the nervous system, and cancer between pre-treatment and post-treatment groups, suggesting that GQD changed the functional state of patients with CRC via the gut microbiome [40].

There was no difference in the proportion of peripheral immune cells between the control and pre-treatment groups. However, the post-treatment group showed a significant increase in cluster of differentiation (CD) 4^+^ T cells and Natural Killer T (NKT) cells compared to both other groups. Other immune cell types (CD8^+^ T, Natural Killer (NK), and T regulatory (Treg) cells) didn’t present a significant difference between the three groups [40]. In the post-treatment group, Tumor Necrosis Factor (TNF)-α levels were significantly lower when compared to the control and pre-treatment groups. Additionally, GQD also reduced the level of 5-hydroxytryptamine (5-HT) levels compared to pre-treatment values, although the reduction compared to the control group was not statistically significant. No significant changes were observed in interferon (IFN)-γ, (interleukin) IL-2, IL-6, or IL-10 levels between the three groups [40]. In terms of the distribution of lymph nodes and destruction of intestinal mucosa, the control group presented a more severe inflammatory reaction than the treatment group. The treatment group showed significantly higher expression of Zonula Occludens (ZO)-1 in both tumor and normal tissues. Similar trends were observed for occludin and nuclear factor kappa B (NF-κB) in tumor tissues: occludin was upregulated and NF-κB downregulated in the treatment group, while there was no difference in normal tissues between control and treatment groups [40]. In the treatment group, 22% of the patients (n = 2) experiencing stomachache/bloating reported symptom alleviation. Symptomatic improvement was observed in 86% (n = 12) of the patients with diarrhea and in 58% (n = 7) of those presenting tenesmus [40]. However, because the study lacked a placebo control, participants knew their treatment status, which may have influenced the reliability of the reported subjective adverse event.

Overall, these findings suggest that GQD exerts its therapeutic effects as a multifaceted herbal modulator of the gut microenvironment, particularly by increasing the abundance of SCFA-producing bacteria. These, in turn, may influence host physiology, supporting immune balance, barrier restoration, and reduced intestinal inflammation. However, the certainty of these findings may be compromised by the selection of the reported results bias, as this study did not follow a publicly available pre-specified protocol.

### 3.4 Host Factor Supplementation: Vitamin D

Finally, Bellerba et al. [41] conducted a phase II clinical trial involving CRC patients to evaluate the impact of vitamin D supplementation on gut microbiome composition and to assess whether the microbiome may mediate serum 25-hydroxyvitamin D [25(OH)D] levels. Vitamin D is well-recognized for its immunomodulatory and anti-carcinogenic properties [64]. In contrast with the previous interventions presented, such as standard CRC treatments and direct microbiota-targeting interventions, vitamin D and its receptor (VDR) can indirectly affect microbiome composition, and thereby influencing immune responses, intestinal barrier integrity, and inflammatory processes [65].

All participants had completed standard treatment (surgery with or without chemotherapy and/or radiotherapy) and, at the time of enrolment, showed no evidence of active neoplasia. Seventy-four patients were stratified according to their prior adjuvant or neoadjuvant therapy and then randomized into either a placebo group (n = 32) or a supplementation group (n = 28). The intervention lasted one year. Blood and fecal samples were collected simultaneously at two distinct times: baseline and after twelve months (M12) [41].

The supplementation group was predominantly characterized by the presence of the *Bacteroides* genus. At the species level, it was also characterized by *F. prausnitzii* and *Holdemanella biformis*, and showed higher abundances of *Leuconostoc pseudomesenteroides, Bacteroides gallinarum, Christensenella timonensis*, and *Ruminococcus YE78*. In contrast, the placebo group was associated with *Shigella boydii* and *Raoultella ornithinolytica*, as well as various species from the *Streptococcus* and *Escherichia* genera [41]. Independent of treatment arm, patients who achieved vitamin D sufficiency exhibited increased abundances of *Leuconostoc pseudomesenteroides, Ruminococcus YE78, F. prausnitzii*, and *Bacteroides clarus*. Conversely, *Eubacterium brachy* and *Bacteroides coprocola* were more prevalent among placebo-treated patients and those who did not reach vitamin D sufficiency by the end of the study [41]. Vitamin D supplementation directly increased the final 25(OH)D levels and modulated a subgroup of taxa, which indirectly affected the final 25(OH)D levels [41].

An inverse correlation was observed between *F. nucleatum* abundance and post-treatment 25(OH)D levels. Notably, baseline, but not post-treatment, presence of *F. nucleatum* was significantly associated with poorer disease-free survival (DFS). Given the limited follow-up duration of the trial, clinical events were defined to include the occurrence of colorectal adenoma, cancer relapse, or death. Additional risk factors were identified in relation to *F. nucleatum* abundance and recurrence risk. Baseline body mass index (BMI) was inversely correlated with the risk of recurrence. Women were significantly more likely than men to have *F. nucleatum* at the end of treatment, regardless of its presence at baseline. Among those positive for *F. nucleatum* post-treatment, abundances were inversely correlated with age, higher in those already carrying the bacterium at baseline, and marginally lower in those who reached vitamin D sufficiency [41].

Functional metagenomic analyses revealed that several microbial pathways were significantly enriched in vitamin D-supplemented individuals, including D*-fructuronate degradation*, *acetyl-CoA fermentation to butanoate II*, the *superpathway of glycerol degradation to 1,3-propanediol*, the *superpathway of thiamine diphosphate biosynthesis II*, and *guanosine nucleotide degradation II*, with the latter three pathways also more abundant in vitamin D-sufficient individuals’ post-treatment. In contrast, *L-histidine biosynthesis* and *pyrimidine deoxyribonucleoside salvage pathways* were more abundant in placebo-treated patients, while *L-ornithine de novo biosynthesis* was more prevalent among those with persistent vitamin D deficiency [41].

Interestingly, an interaction between sex and vitamin D levels was also observed, as it was revealed that men and women differed in microbial taxa composition at follow-up, which in turn influenced final 25(OH)D levels. While no sex-based differences in metabolic pathway abundance were observed in the placebo group, a significant divergence emerged in the supplementation group. Specifically, the *superpathways of L-lysine, L-threonine, and L-methionine biosynthesis II*, as well as *L-histidine biosynthesis*, were significantly more abundant in women than in men following supplementation. However, these differences were not evident among non-supplemented participants [41].

Overall, vitamin D supplementation led to a direct increase in serum vitamin D levels and influenced a specific subset of microbial taxa, which may have contributed indirectly to the final vitamin D levels. These findings suggest a reciprocal relationship between vitamin D levels and gut microbiome composition, potentially contributing to the beneficial effects in CRC patients.

The synthesis of microbiome variations together with the corresponding clinical outcomes for each therapeutic regimen in CRC patients is summarized in Table 3. To complement these findings, the certainty of evidence across outcomes, assessed using the GRADE framework, is presented in the SoF table (Table 4).

**Table 3:** The table presents the variations of microbiome composition and clinical outcomes of the studies included in this review. The information compiled includes intervention, microbiome composition and clinical outcomes

Study	Intervention	Microbiome composition	Clinical outcomes
Žukauskaitė et al., 2024 [36]	The OP group ingested 4 L of Macrogol 4000 (73.69 mg/L) starting the afternoon before surgery. The RE group was given 2 L of 0.9% sodium chloride. Both groups received antibiotic prophylaxis 30–60 min before surgery (2 g of Cefazolin and 500 mg of Metronidazole).	Between groups, there was no difference in α-diversity and β-diversity on POD6 or POD30. Transient dysbiosis in both groups. Persistent shifts: OP ↓ Porphyromonas ↑ Collinsella, Eubacterium halli, Eubacterium coprostanoligenes RE↑ *Actinomyce*s, *Enterococcus, Parabacteroides*	No difference in postoperative morbidity rates and severity of complications between groups. Those who developed infections showed higher abundance on POD6 of bacteria from the *Actinomyces* genus, *Sutterella* uncultured species, and *Enterococcus faecalis* species.
Aarnoutse et al., 2022 [37]	Three cycles of Capecitabine (1000–1250 mg/m^2^, twice daily on days 1–14 in a 3-week cycle) with or without Bevacizumab (7.5 mg/kg on day 1 every 3 weeks).	No significant differences for α-diversity and β-diversity between R and NR	Significant inter-individual heterogeneity during treatment, but no prominent capecitabine-induced pattern. NR showed higher grades of fatigue compared to R.
Sánchez-Alcoholado et al., 2021 [38]	Five weeks of Neoadjuvant Radiochemotherapy with Pelvic Radiation Therapy (50 Gy in fractions of 2 Gy/session) and Capecitabine (825 mg/m^2^/12 h), followed by surgical resection.	No significant differences for α-diversity and β-diversity during neoadjuvant treatment. R presented a higher diversity and richness than NR. R:↑ *Bifidobacterium bifidum, Ruminococcus albus, Faecalibacterium prausnitzii* NR:↑ *Prevotella copri, E. coli, Fusobacterium nucleatum, Bacteroides fragilis*	Responders exhibited greater microbial diversity with distinct composition and activity. R had increased concentrations of SCFA, whereas NR showed increased polyamines and zonulin levels.
Huang et al., 2023 [39]	Both groups underwent surgical resection followed by the first cycle of adjuvant Xelox Intervention group took one capsule of probiotic (0.5 g and contained over 0.5 × 10^6^ CFU of *B. infants*, *L.acidophilus*, *E. faecalis*, and over 0.5 × 10^5^ CFU of *B. cereus*) 3 times per day from the third postoperative day to the end of the first chemotherapy course, while Placebo group took placebo tablets.	Treatment regimen disturbs α-diversity and microbial composition, but probiotic supplementation reverses this trend. Placebo: ↑ *Akkermansia, Ruminococcus, Enterococcus*	Probiotic supplementation reshapes gut bacterial populations and is correlated with the production of SCFA. Probiotic supplementation does not affect the antitumor efficacy of chemotherapy. Probiotic administration effectively reduces some chemotherapy-induced gastrointestinal complications (abdominal pain, abdominal distention, constipation, diarrhea).
Li et al., 2020 [40]	The Control group received routine treatment followed by surgery. The Intervention group receives routine treatment and 250 mL (twice daily) of GQD (*Radix Pueraria*e (15 g), *Scutellariae Radix* (9 g), *Coptidis Rhizoma* (9 g), and *Liquorice* (6 g)) for 7 days, followed by surgery.	α-diversity and β-diversity were significantly reduced in the post-treatment group. Intervention: ↑ *Bacteroide, Akkermansia, Prevotella* ↓ *Megamonas, Veillonella*	Intervention implicates a microbial shift, which reflects functional differences in the pathways associated with energy metabolism, immune regulation, neurological function, and cancer. GQD increased the proportions of CD4^+^ T and NKT cells and reduced TNF-α and 5-HT levels in the post-treatment group. In tumor tissues from the treatment group, occludin expression was significantly elevated, while NF-κB and TNF-α levels were reduced. ZO-1 expression significantly increased in the tumor and the adjacent normal tissues. In the treatment group, 22% of patients experiencing stomachache/bloating reported symptom alleviation. Symptomatic improvement was observed in 86% of patients with diarrhea and 58% of those presenting with tenesmus.
Bellerba et al., 2022 [41]	Supplementation of Vitamin D_3_ 2000 IU or placebo once daily for one year.	Supplementation: ↑ *Faecalibacterium prausnitzii, Holdemanella biformis, Bacteroides gallinarum* Vitamin D sufficiency: ↑ *Leuconostoc pseudomesenteroides, Ruminococcus YE78, Faecalibacterium. prausnitzii, Bacteroides clarus* Placebo: ↑ E*ucobacterium branchy, Bacteroides coprocola* An inverse correlation was observed between *F. nucleatum* abundance and post-treatment 25(OH)D levels	Vitamin D supplementation directly increased the final 25(OH)D levels and modulated a subgroup of taxa, which indirectly affected the final 25(OH)D levels. Vitamin D supplementation reshapes gut microbial function, favoring pathways linked to energy metabolism, vitamin biosynthesis, and anti-inflammatory SCFA production. Presence of *F. nucleatum* at baseline, but not post-treatment, was significantly associated with poorer DFS.

Note: 5-HT, 5-hydroxytryptamine; CD, Cluster of Differentiation; DFS, Disease Free-Survival; GQD, Gegen Qinlian Decoction; Gy, Gray; NF-kB, Nuclear Factor kappa B; NKT, Natural Killer T; NR, Non-Responders; OP, Osmotic diarrhea-inducing Oral Preparation; POD, Postoperative Days; R, Responders; RE, Rectal Enema; SCFA, Short-Chain Fatty Acids; TNF-α, Tumor Necrosis Factor α; ZO-1, Zonula Occludens-1; ↑, Increased Abundance; ↓, Decreased Abundance. Underlined text indicates the specific components of the therapeutic regimens, as well as the specific groups being compared.

**Table 4:** The SoF table presents the certainty of evidence across outcomes. The information compiled includes outcomes, number of participants, study design, the five domains in GRADE, and overall certainty of the findings

Outcomes	Number of participants	Study design	Risk of bias	Inconsistency	Indirectness	Imprecision	Publication bias	Overall certainty
Clinical response to therapy	233	2 RCT, 2 Observational Studies	No serious limitations (a)	Serious (b)	Serious (c)	Serious (d)	Undetected (e)	Low
Treatment-related toxicity/adverse effects	241	3 RCT, 1 Observational Study	Serious limitations (f)	No serious	No serious	Serious (d)	Undetected (e)	Low
Gut microbiome indices	291	3 RCT, 2 Observational Studies	No serious limitations (a)	Serious (b)	Serious (c)	Serious (d)	Undetected (e)	Low
Taxonomic composition	341	4 RCT, 2 Observational Studies	No serious limitations (a)	Serious (b)	Serious (c)	Serious (d)	Undetected (e)	Very low
Microbial metabolites and biomarkers	200	3 RCT, 1 Observational Study	No serious limitations (a)	Serious (b)	Serious (c)	Very serious (d)	Undetected (e)	Very low

Note: RCT, Randomized Clinical Trial. (a) Minor concerns in RCTs and inherent confounding in observational studies but generally acceptable designs; (b) Heterogeneous diversity and microbial findings across studies with some distinct associations; (c) Outcomes relied largely on surrogate microbiome measures (diversity, taxa, metabolites) and varied CRC stages and therapeutic regimens; (d) Small sample sizes and wide uncertainty across effect estimates; (e) Too few studies to detect potential bias and no obvious selective reporting observed; (f) Concerns regarding the reliability of subjective, self-reported adverse events.

## 4 Discussion

The gut microbiome constitutes a natural defense, and it’s involved in numerous protective, structural and metabolic functions, playing a notorious role in maintaining gut homeostasis [7,8]. Even though it is not clear whether dysbiosis is a cause or a consequence of CRC, the gut microbiome seems to have an important role in CRC pathogenesis [10–13].

It’s currently fairly accepted that CRC patients present a dysbiotic gut microbiome composition when compared to healthy individuals [7,66]. Most studies included in this review reference this, with some fair agreement between them. The composition and activity of the gut microbiome are then further altered by cancer treatments, regardless of which kind [8,14].

### 4.1 Microbiome Composition across Studies

In general, there’s a consensus across the studies that better responsiveness to treatment is directly related to modulation of the gut microbiome, enriched in potentially protective taxa and decreased pro-carcinogenic ones, leading to alteration in immune response, gut barrier integrity and response to inflammation.

In the gut microbiome composition, there was a notable increase in traditional probiotic taxa, such as *Lactobacillus* and *Bifidobacterium*. These genera have exhibited anticancer effects in preclinical models through diverse mechanisms, including suppression of cell proliferation, induction of cancer cell apoptosis, modulation of immune response, inactivation of carcinogenic toxins, and synthesis of anticarcinogenic metabolites [8]. In the included studies, both are associated with better response in Sánchez-Alcoholado et al. study [38], while also being increased after probiotic supplementation in Huang et al. study [39]. These genera may also influence physiological dysfunctions associated with fatigue, potentially mitigating its effects, although the underlying mechanisms remain unclear [67]. In Aarnoutse et al. study [37], although no specific bacterial taxa could be identified for Responders and Non-Responders, an increased abundance of these genera may be linked to reduced fatigue in the Responders group compared to the Non-Responders group.

Simultaneously, there is a noted increase in some butyrate-producing genera, such as *Roseburia* and *Faecalibacterium*, which are usually depleted in CRC patients. Particularly, *F. prausnitzii* is a known gut commensal; it is increased both in Bellerba et al. Vitamin D supplementation group [41], while also being associated in Sánchez-Alcoholado et al. with the R group [38]. *F. prausnitzii* is a potential probiotic, producing microbial anti-inflammatory molecules that can downregulate the NF-kB pathway in intestinal epithelial cells and prevent colitis in animal models [68].

On the other hand, the *Fusobacterium* genus shows concomitantly reduced after probiotic supplementation and is associated with non-responsiveness in Sánchez-Alcoholado et al. study [38]. *F. nucleatum*, in particular, is one of the most extensively studied strains in CRC development. It disrupts barrier function, promotes inflammation by modulating the tumor microenvironment, and activates pro-oncogenic signaling pathways that support CRC progression [69]. In the study by Bellerba et al., an inverse correlation was observed between the abundance of this species and post-treatment vitamin D levels [41]. However, when assessing its association with DFS, only baseline levels of *F. nucleatum* were linked to worse DFS. Such an association was not found post-treatment, which the authors suggested that *F. nucleatum* may serve more as a marker of patient health status than as an active driver of tumorigenesis.

Finally, *Enterococcus* genus shows be increase in placebo group of Huang et al. study [39] and its presence, specifically *E. faecalis*, seem to be related to adverse events in Žukauskaitė et al. study [36]. *E. faecalis* is a gut commensal bacterium and is typically enriched in CRC patients [70]. The mechanisms linking *E. faecalis* to colorectal carcinogenesis remain unclear; however, it has been shown that this species produces pro-oxidative reactive oxygen species (ROS) and enterotoxins, as well as presenting collagen-degrading activity, which can lead to oxidative DNA damage, induce inflammation, and damage the epithelial barrier [71–73]. This is further supported by Žukauskaitė and colleagues, who identified *E. faecalis* levels increasing significantly on POD6 in individuals who presented postoperative complications, while those confirmed to be caused by this species showed a concurrent increase in abundance [36].

However, despite these shared patterns, individual studies revealed notable divergences. The *Akkermansia* genus, while it was found enriched after GQD supplementation in Li et al. study, Huang and colleagues appointed it as increased in the placebo group when compared to pre-treatment. *A. muciniphila* extracellular vesicles can enhance intestinal barrier function by regulating occludin via AMP-activated protein kinase (AMPK) activation [74]. Conversely, *A. muciniphila* may become detrimental in certain intestinal disease contexts, where its excessive mucin-degrading activity [75] can compromise the mucosal barrier [76].

In the study by Li et al., an increase in the *Bacteroides* genus was observed following GQD supplementation [40], while Sánchez-Alcoholado and colleagues associated this genus with the Non-Responders group [38]; notably, *B. fragilis* was identified as a specific biomarker of the Non-Responders group. In particular, Enterotoxigenic *B. fragilis* (ETBF) has been implicated as a cancer-promoting bacterium. Murine models have demonstrated that ETBF, through its toxin, initiates an inflammatory cascade in colonic epithelial cells by activating the Th17 immune response, creating a pro-inflammatory environment [77,78]. However, *Bacteroides* also seems to play an important role in modulating the human immune system by metabolizing polysaccharides and oligosaccharides [79]; for instance, *B. vulgatus* has been shown in murine models to reduce colitis-associated colorectal tumors [80]. *B. clarus* is usually reduced in CRC patients [81], while Bellerba and colleagues showed that Vitamin D sufficiency may increase this species [41].

The *Prevotella* genus was found to be enriched following GQD supplementation in the study by Li et al. and after probiotic intervention in Huang et al. study [39,40], yet it was also more abundant in the Non-Responders group in the study by Sánchez-Alcoholado et al. [38]. Consistently, *Prevotella* tends to be enriched in CRC patients, potentially contributing to pathogenesis by modulating the expression of immunoinflammatory response genes [82,83]. In contrast, murine models have shown significantly lower levels of this genus in CRC-bearing rats compared to healthy controls, suggesting a possible inverse association between *Prevotella* abundance and CRC progression [84].

The *Ruminococcus* genus was associated with the Responders group in Sánchez-Alcoholado et al. study and vitamin D sufficiency in Bellerba et al. study [38,41], whereas Huang et al. observed an increase in placebo group [39]. *Ruminococcus* is a butyrate-producing genus typically found to be depleted in CRC patients [82,85]. Several strains have been associated with potential benefits in CRC. In murine models, *R. gnavus* has been shown to metabolize lysoglycerophospholipids and support the immune surveillance function of CD8^+^ T cells [86]. *In vitro* studies have identified *R. bromii* as a key contributor to colonic butyrate production [87].

### 4.2 Functional Characteristics of the Microbiome

Such microbial shifts are not incidental but carry important functional consequences. As mentioned before, it is acknowledged that the gut microbiome has a significant impact not only on gut homeostasis, directly by metabolic byproducts, but also indirectly, modulating immune response, altering epithelial barrier and managing inflammation.

One of the most relevant capacities of the gut microbiome is to ferment complex carbohydrates, generating metabolites such as SCFA, principally butyrate, acetate and propionate [88]. SCFA have a key role as metabolic and immune mediators, often depleted in CRC patients [89]. As explored in the included studies, better response and decreased adverse effects have seemed to be correlated to an enrichment of SCFA-producing taxa.

In Sánchez-Alcoholado et al. [38], besides the enrichment of *Bifidobacterium* and *Roseburia* in Responders group, it is identified specific species which are directly correlated with increased SCFA: in Responders group, *F. prausnitzii* and *R. albus* are positively associated with increased butyric and acetic acids, while in Non-Responders group, it seems that *B. fragilis* the responsible for the production of propionate. Furthermore, Bellerba et al. [41] notes increased levels of *F. prausnitzii* in all who reached vitamin D sufficiency.

In Huang et al. [39], accordingly, is also seen between increased *Lactobacillus* and *Roseburia* and increased SCFA in Probiotic group. As stated before, *Bacteroides* also seems to play an important role in modulating the human immune system by metabolizing polysaccharides and oligosaccharides, being enriched both in Li et al. [40] after GQD intervention and individuals with vitamin D sufficiency in Bellerba et al. [41].

Changes in gut microbial metabolites may have a wide range of effects on CRC pathogenesis [90]. SCFA appear to play an important role in regulating the integrity of the epithelial barrier through coordinated regulation of tight junction proteins (TJP), specifically through the increased expression of ZO-1 and occludin [91]. It also seems that SCFA decreases bacterial lipopolysaccharide (LPS) translocation, inhibiting a toll-like receptor 4 (TLR4) triggering and, consequently, precluding activation of signaling pathways such as NF-kB and inflammation driven by cytokines such as TNF-α [92], thus modulating the immune response and contributing to the reduction of inflammation.

The GQD intervention is related to an increased abundance of *Bacteroides* and *Prevotella*, both recognized as propionate-producing genera [93,94], which is and in line with what was previously explained about the mechanism of action of SCFA. It is also observed that an increase in *Akkermansia* genus. This genus has been associated with enhancing mucus layer thickness and repairing gut barrier damage [95]. Specifically, *A. muciniphila* produces SCFA [96], which may contribute to the mechanisms described above.

On the other hand, Sánchez-Alcoholado et al. [38] study depicted *Prevotella copri* in NR group, which was associated with increased zonulin levels. Animal models previously showed a correlation between these species and increased host intestinal barrier permeability markers, such as zonulin, as well as activation of host chronic inflammatory responses [97]. This restoration of gut barrier integrity, as well as suppression of inflammation, could be a key factor in the reduction of treatment-related adverse effects, such as diarrhea [98], as documented in both Huang et al. [39] and Li et al. [40] studies.

Dysbiosis can activate dendritic cells (DC), initiating a protective immune response through Th1 and Th17 cell polarization. Conversely, the gut microbiome and its byproducts can also promote Treg cell activity, which modulates Th1 and Th17 responses [99]. In the context of CRC, the balance between Th1 and Th17 cells appears to be critical: increased Th1 responses are associated with improved outcomes, whereas elevated Th17 activity correlates with poorer prognosis [100].

Among SCFA, butyrate plays a particularly significant role by activating the aryl hydrocarbon receptor (AhR), which leads to the downregulation of the pro-inflammatory Th17 response, inhibiting inflammation and thereby contributing to gut homeostasis [101,102]. AhR is a transcription factor that promotes xenobiotic metabolism, which is noted to be one of the pathways associated with R group in Sánchez-Alcoholado et al. [38] study, further highlighting a potential mechanistic connection between microbial metabolites and immune modulation.

Li et al. associate GQD supplementation with increased CD4^+^ T cells and NKT cells in peripheral blood through modulation of the gut microbiome [40]. Even though not fully stated, it could be understood that the increased concentration of SCFA-producing bacteria is the reason for this. It is understood that SCFA contribute to anti-tumor immunity by promoting the infiltration of T cells, such as CD4^+^ T cells, into the tumor microenvironment and activating T cell-mediated immune responses [103]. This further explains how the microbiome post-treatment presented functional differences in immune system.

NKT cells serve as central regulators of both intestinal and tumor immunity by interacting with DC, NK cells, CD4^+^ T cells, and CD8^+^ T cells [104]. Their functional role, either pro- or anti-tumor, is influenced by their subtype and the surrounding microenvironment. Type I and type II NKT cells are known to cross-regulate each other, and murine studies suggest that, in the absence of this regulation, Tregs become the primary modulators of tumor immunity, fostering an immunosuppressive microenvironment [105]. Moreover, microbial dysbiosis has been shown to reduce NKT cell populations while expanding Treg, which conversely can further impair NKT cell function [106].

Although not yet fully understood, 5-HT levels are known to be significantly upregulated in CRC patients [107]. While current evidence supports a regulatory role for 5-HT, its associated mechanisms can also shift toward procarcinogenic activity [108]. Emerging studies suggest that the gut microbiome influences the production and homeostasis of enteric 5-HT, potentially through the action of SCFAs [109]. As such, Li and its colleagues associated decreased 5-HT levels to the gut microbiome [40].

In addition to SCFA, other microbial metabolites, such as ROS, can significantly influence immune responses, intestinal barrier integrity, and inflammation. While ROS act as signaling molecules involved in cell growth, differentiation, and immune regulation [110], their overproduction in the context of a dysbiotic microbiome can promote DNA damage and chromosomal instability, contributing to inflammation and epithelial barrier disruption [82,111]. Both *E. faecalis* and *F. nucleatum* have been implicated in ROS production [99]. In Sánchez-Alcoholado et al. study [38], the increased abundance of *F. nucleatum* in the NR group may explain the activation of the oxidative phosphorylation pathway.

Polyamines are small molecules synthesized from amino acids such as arginine and ornithine by both host tissues and the gut microbiota [11]. They play essential roles in maintaining intestinal barrier integrity, regulating cell proliferation, modulating immune cell differentiation, and exerting anti-inflammatory effects [112–114]. However, their impact on cancer is context-dependent, as polyamines can be both anti-carcinogenic and pro-tumorigenic depending on their concentration, metabolic derivatives, tumor stage, and therapeutic context.

In the host, polyamine biosynthesis involves arginase 1 converting L-arginine to L-ornithine, followed by ornithine decarboxylase (ODC) synthesizing putrescine. Subsequent enzymes interconvert putrescine, spermidine, and spermine [115]. In CRC, polyamine upregulation, mainly driven by ODC, is associated with cell dysfunction and tumorigenesis [25].

Polyamines also interact with the gut microbiome in tumor-promoting ways. Host-derived polyamines like N1, N12-DiAcSP can promote bacterial biofilm formation, which in turn enhances microbial polyamine production and further supports cancer progression [116].

Pathogens such as *E. coli* and ETBF contribute by producing polyamines; ETBF generates ROS, leading to inflammation and DNA damage, while colibactin-producing *E. coli* actively produces spermidine to present genotoxic activity [117,118]. In Sánchez-Alcoholado et al. study, it is noted an increased abundance of *E. coli* in the Non-Responders group may reflect this mechanism [38]. These findings may also explain the reported positive correlation between *B. fragilis* and elevated levels of N1, N12-DiAcSP and N8-AcSPD in the same group [38].

Additionally, tumor cells exhibit increased polyamine levels to support rapid proliferation [119]. Elevated polyamine metabolism enables DC function in suppressing immune responses [25]. Polyamine-blocking therapies have shown promise in inhibiting tumor growth and enhancing programmed death-1 (PD-1) immunotherapy [120]. Notably, spermine accumulation has been linked to promote dysbiosis in murine models [121].

Finally, amino acids can serve as alternative precursors for SCFA synthesis by gut microbiome [122]. A shift toward bacterial protein fermentation was related to high colonic pH and low carbohydrate availability [112], which suggests that in the CRC context, depletion of the SCFA-producing bacteria leads to activation of other metabolic pathways, such as amino acids.

Sánchez-Alcoholado and colleagues refer there’s a negative correlation between *F. prausnitzii* and N1, N12-DiAcSP and spermidine, which could be explained by this mechanism [38]. With the increase in SCFA-producing bacteria, there’s a downregulation of amino-acid metabolism, leading to a decrease in these polyamines. This is further suggested by Bellerba et al., who identified “*L-ornithine de novo biosynthesis*” as more prevalent in individuals with persistent vitamin D deficiency; those individuals also decrease in SCFA-producing bacterium, which would explain the prevalence of an amino-acid pathway [41]. Interestingly, both studies appoint enriched more metabolically versatile pathways to distinct groups; while Sánchez-Alcoholado and colleagues noted that NR group shows enriched genes related to amino acids, lipid and vitamins metabolic pathways, those same pathways are more prevalent in vitamin D sufficiency in Bellerba et al. study [38,41].

### 4.3 Differences across Microbiome Compositions and Functional Redundancy

However, it wasn’t found a universally gut microbiome pattern among all the individuals. A major point of difference is the inconsistent changes in α-diversity and β-diversity across studies.

The Žukauskaitė et al. [36] study reports no significant changes in both α-diversity or β-diversity between interventions, even though it found significant compositional shifts within-group through time. Both Aarnoutse et al. [37] and Sánchez-Alcoholado et al. [38] couldn’t find a significant change in α-diversity over time during standard treatment. However, when comparing Responders and Non-Responders groups, Sánchez-Alcoholado and colleagues showed that R group presented higher diversity, richness and a significant shift in microbiome composition, while Aarnoutse and colleagues were unable to identify α-diversity or β-diversity fluctuations.

Current literature implies that CRC patients present reduced diversity and richness compared to healthy individuals [123], which is then further altered by cancer treatments [8,14]. It is then uncommon the usableness to identify α-diversity variations. However, it is important to note that in Sánchez-Alcoholado et al. [38] study, when patients were stratified by therapy response, Responders group exhibited higher α-diversity and distinct microbial composition (β-diversity). This suggests that stable diversity metrics may still mask biologically meaningful compositional shifts in specific patient subgroups.

When introducing Huang et al. [39], Li et al. [40] and Bellerba et al. [41] studies, it gets further clearer how specific interventions lead to distinct microbial impacts. Huang et al. [39] indicates that XELOX regimen preceded by surgery reduces α-diversity, which is then maintained by probiotics; according to the analysis of gut microbial β-diversity, there’s some fluctuation over time, unrelated to intervention, although there were no clear distinctions for the fecal microbial communities among all groups. Li et al. [40] explicitly state a reduction in both α-diversity and β-diversity after GQD treatment. Finally, Bellerba et al. [41] show a clear distinction in gut microbiome composition between treatment arms.

These divergences in gut microbiome constitution are further exposed when analyzing differentially abundant taxa in individuals. As stated before, some genera seem to be globally associated with better or worse outcomes, while others seem to be a “double-edged sword”. However, and as stated before, there’s a consensus across the studies that better therapeutic outcomes and decreased adverse effects are related to enriching in potentially protective taxa and decreasing pro-carcinogenic ones. These could possibly be explained by the existence of a functional redundancy: microbiomes that differ in terms of composition may share functional mechanisms, yielding similar protein or metabolite profiles [124]. For instance, Sánchez-Alcoholado et al. reported an enrichment of the *Faecalibacterium* genus, particularly *F. prausnitzii*, in the R group, correlating with increased butyric acid [38]. In contrast, Huang et al. did not report *Faecalibacterium*, but instead identified *Roseburia*, another well-established butyrate-producing genus, as enriched in association with elevated SCFA [39]. Despite taxonomic differences, both studies suggest a convergent functional outcome: enrichment of SCFA-producing bacteria. Multiple factors can explain these variations in gut microbiome compositions.

### 4.4 Exogenous and Endogenous Factors as Risk Drivers

CRC is increasingly recognized as a heterogeneous disease that arises from the interplay of exogenous and endogenous factors, each of which interacts with the host microenvironment to shape disease pathogenesis and treatment response.

There’s a wide range of etiologic factors for CRC. Exogenous factors include diet, lifestyle habits such as physical activity and smoking, alcohol consumption, medications, and microbial or viral infections. Endogenous factors encompass age, sex, BMI, family history of CRC, host genetics, and epigenetic predispositions [7,125–127]. Together, these factors can influence the tumor microenvironment, immunity and cellular signaling [128–130], which can either preserve tissue homeostasis or promote malignant transformation.

A critical aspect of this heterogeneity is that these exposures do not act in isolation; instead, they converge on key cellular processes, including epigenetic regulation, metabolic rewiring, inflammation, and immune surveillance [129–133]. While diet may alter intestinal cell signaling pathways [134], smoking can promote epigenetic changes [135], and germline predispositions influence host responses to pathogenic development [133]. In this way, risk factors function less as linear determinants of cancer and more as facilitators of specific pathogenic processes.

### 4.5 Disease Heterogeneity and Molecular Pathological Epidemiology (MPE)

As stated before, CRC is a heterogeneous group of neoplasms, uniquely influenced by a complex interplay of endogenous and exogenous factors and their interactions with both normal and dysfunctional cells, leading to distinct molecular alterations in each individual [129,133].

Molecular pathological epidemiology (MPE) provides a framework to integrate these multidimensional influences. It is built on two paradigms: the “Unique Tumor Principle”, which holds that each cancer arises through a distinct pathologic process despite shared features, and the “Disease Continuum Theory”, which posits that different diseases can share overlapping etiologies and pathogenic mechanisms [136].

In this lens, the MPE acknowledges CRC heterogeneity and the complexity of pathogenic mechanisms by linking exposures to molecular signatures, enabling the identification of exposure–pathway–outcome relationships that explain why patients with seemingly similar cancers can have markedly different clinical trajectories [137].

Although MPE emphasizes the unique characteristics of each tumor, it also posits that subgrouping disease by shared molecular characteristics can help predict, to some extent, its evolution and progression [129]. On this line, the Consensus Molecular Subtype (CMS) classification of CRC clearly exemplifies this. This framework defines four subtypes with unique molecular and clinical features.

CMS1 (Microsatellite Instability (MSI)-immune) is associated with activation of the *Janus kinase/signal transduction and transcription* (JAK-STAT) signaling pathway, MSI and CpG Island Methylator Phenotype (CIMP) phenotypes, proto-oncogene *B-Raf* (BRAF) mutations and the serrated pathway of carcinogenesis [126,138,139]. CMS2 (Canonical subtype) is linked to high levels of somatic copy number alterations (SNCA) and chromosomal instability (CIN), which is a hallmark of cancers arising from the adenoma-carcinoma pathway, as well as associated with the activation of the *Wingless-related integration site/myelocytomatosis oncogene* (Wnt/Myc) signaling pathway [126,138,139]. MS3 (Metabolic) reflects metabolic dysregulation and *Kirsten rat sarcoma virus* (KRAS) mutations, often expressing a mixed MSI phenotype and includes both low SCNA and CIMP tumors [126,138–140]. CMS4 (Mesenchymal) is also characterized by high SCNA, indicative of CIN, with the tumors presenting strong *Transforming growth factor* (TGF)-β activation related to immunosuppression, epithelial-mesenchymal transition (EMT) and angiogenesis [126,138].

Broadly, CMS1 aligns with the serrated pathway, while CMS2–4 correspond to subtypes of the CIN pathway, whilst allowing more detailed characterization of the molecular genetics. The serrated pathway, and thus CMS1, is marked by BRAF mutations, DNA methylation abnormalities [141] and increased CIMP, which silences tumor suppressor genes and can lead to MSI [142]. Findings suggest that CIN and CIMP are independent and inversely related mechanisms of genomic instability in sporadic CRC [142]. Within an MPE framework, the association of CIMP-high with older age, female sex, smoking, and proximal tumor location suggests that these factors may predispose to CMS1 tumors [135,143–145]. Obesity, on the other hand, may favor CMS3. Excess energy and nutrients provided by obesity can fuel tumor growth [146], with fatty acid synthase (FASN) supporting lipid biosynthesis in rapidly dividing cells. Normally, AMPK inhibits FASN under energy stress [147], but obesity allows FASN-driven metabolism to persist [148]. Since obesity is linked to cancers lacking active WNT–CTNNB1 signaling [130], it may be hypothesized that it could favor CMS3 development at the expense of CMS2, driving carcinogenesis through metabolic dysregulation rather than canonical WNT–β-catenin activation. This integrative approach is essential for understanding how a wide range of factors, acting in concert with genetics, produce clinically meaningful heterogeneity.

### 4.6 The Microbiome as a Key Interacting Factor

The gut microbiome has emerged as a central determinant of heterogeneity in CRC. When focusing on Microbiology-MPE, it provides a promising approach to explore the interpersonal heterogeneity of the carcinogenic process in relation to the altered microbial composition and to understand how distinctive phenotypes of tumors arise in the presence of specific microorganisms [130]. For instance, certain bacterial species appear to drive specific molecular pathways [149].

For example, *Fusobacterium* species, particularly *F. nucleatum*, are associated with CMS1 [150] and influence immune responses by recruiting CD11b+ myeloid cells that differentiate into macrophages, granulocytes, and dendritic cells, thereby promoting tumor growth and immune suppression within the tumor microenvironment [151]. While CMS2 tumors seem to harbor low bacterial biomass, increased abundances of *Selenomonas* and *Prevotella* have been observed in some cases [150,152]. Microbial metabolites also play a role: formate, produced by *F. nucleatum* and others, can activate AhR signaling, enhancing cancer stem cell proliferation, migration and xenobiotic metabolism, features that may align with CMS3 [153–155]. Similarly, *B. fragilis* toxin activates WNT signaling via β-catenin nuclear accumulation, fostering a mesenchymal phenotype and proliferation, potentially contributing to CMS4 [156–159]. These findings underscore that the microbiome is not merely a secondary modifier of disease but an active driver of tumorigenic pathways and molecular subtypes.

Besides this, and even though it’s understood that distinctly, both exogenous and endogenous factors and microbiome have an intrinsic role in CRC development, there isn’t yet much scope that evaluates how these exogenous and endogenous factors can also possibly alter gut microbiome and, in doing so, what is the impact on both CRC development and response to therapy. Interestingly, Aarnoutse et al. noted intra-individual microbiome alterations during treatment; however, these changes were inconsistent and appeared influenced by external factors [37].

Diet remains one of the most potent external modulators of the gut microbiota [160]. Different studies have shown that certain dietary patterns distinctly shape microbiome composition; notedly, high-fat diets can reduce microbiome diversity and enrich pro-inflammatory bacteria [161]. Importantly, the fermentation of dietary fibers from fruits, vegetables, and whole grains is recommended during cancer therapy, as this process enhances SCFA production [162] and, therefore, provides beneficial effects on gut barrier integrity, inflammation and immune response. Still, the comprehensive effect of overall dietary patterns on microbiome structure remains unclear. Among the studies included in this review, only Sánchez-Alcoholado et al. analyzed the patients’ dietary intake, highlighting a critical variable not evaluated.

Lifestyle behaviors, such as tobacco and alcohol consumption, can also affect gut microbiome composition. Recent studies have shown that CRC patients who smoke or drink exhibit higher intra-individual variability and unstable microbiome composition, indicating their inability to maintain or restore microbiome balance after disruptions [163]. This is speculated to undermine treatment efficacy in these individuals. In Bellerba et al., it is recognized that even without vitamin D supplementation, individuals from placebo group could reach vitamin D sufficiency. The authors further argue that these could be a result of external factors, including diet and increased sunlight exposure [41].

Finally, metabolic health factors, such as BMI, also shape the microbiome composition. It is known that individuals with higher BMI seem to present a more stable microbiome [163], even though this does not equal a healthier profile. The higher-BMI microbiome configuration seems to promote low-grade systemic inflammation, mediated by cytokines such as TNF-α and IL-6, which contribute to tumorigenesis [164]. In contrast with this, Bellerba et al. showed that lower BMI was associated with poorer DFS [41].

Age is another critical factor. Age-related changes in microbiome composition include a gradual decline of both richness and diversity [165]. In line with this, CRC tumors in early-onset CRC (eoCRC) are significantly more diverse and richer when compared to average-onset CRC (aoCRC), even though both presented an overall similar composition of bacterial genera [7]. This suggests that external disruptions may play a key role in eoCRC pathogenesis, further emphasizing the interaction between microbiome and environmental exposures [165]. In the included studies, age does not appear to be a consistently controlled factor. While Žukauskaitė et al., Aarnoutse et al., and Li et al. included individuals aged 18 or older [36,37,40], other studies applied more restricted age ranges: Sánchez-Alcoholado et al. included participants aged between 35 and 75 years [38], Huang et al. between 40 to 70 years [39], and Bellerba et al., 50 to 70 years old [41].

Finally, sexual dimorphism in CRC pathogenesis has also been linked to differential microbiome compositions. Recent studies in murine models have shown that female and male mice exhibited distinct gut microbiome compositions, with the later displaying enrichment of harmful bacteria taxa and depletion of probiotic bacteria when compared with female mice. This microbial imbalance results in altered metabolite production, impacting intestinal epithelial integrity and immune responses, which may contribute to sex-based disparities in CRC treatment outcomes [166]. As shown by Bellerba et al., there’s an interaction between sex, vitamin D levels and gut microbiome composition, with supplementation modulating microbiome composition, which in turn influences final vitamin D levels. It was shown that women had increased pathways related to amino acid production; furthermore, it was also shown that women had a higher probability than men of having *F. nucleatum* at the end of the treatment, regardless of the arm of intervention [41].

Finally, it is increasingly evident that the microbiome plays a critical role in modulating therapeutic response. As highlighted in this review, microbial diversity and specific taxa correlate with treatment efficacy as well as toxicity. In this perspective, and with all stated before, it reinforces the concept that the microbiome has a prevalent role not only as a host exposome, but also as a modulator of tumor biology and therapeutic outcomes.

### 4.7 Beyond Bacteria: Other Elements of Gut Microbiome

Although bacteria dominate most CRC microbiome studies, the non-bacterial microbiome deserves equal attention. Viruses, fungi, and archaea contribute to the complexity of tumor–microbe–host interactions. Although not included in any of the papers included, viruses are an active integrant of the microbiome.

Currently, there are seven viral taxa recognized as human carcinogens. Among them, Epstein–Barr virus (EBV) and human papillomavirus (HPV) have been reported in CRC, alongside cytomegalovirus (CMV) and John Cunningham virus (JCV) [167–169].

EBV can infect B lymphocytes and activate the NF-κB pathway [170]. EBV-infected B cells release microvesicles containing viral molecules that, once internalized by recipient cells, may modulate signaling pathways involved in oncogenesis [171,172]. EBV proteins may also act on microenvironmental and immune cells, altering the tumor microenvironment and potentially supporting CRC initiation or progression.

HPV shows a similarly debated connection with CRC but is thought to promote tumorigenesis by inactivating p53 and modulating the Wnt/β-catenin pathway [173,174]. It may also reprogramme host cell metabolism, which is relevant when discussing that obesity is a CRC risk factor [175–177], and offering a survival advantage in hypoxic conditions, which could facilitate CRC cell persistence, particularly in metastases [178].

CMV, structurally related to EBV but able to infect a wider range of cells [179], has been linked to several cellular factors and alteration and promotion of pro-inflammatory environments that favor DNA damage and oncogenic progression, though its precise molecular roles in colorectal tissue are not fully understood. JCV likewise interferes with the cell cycle, and characteristic mutational signatures in CRC cells suggest it may leave a genomic imprint that contributes to carcinogenesis [180].

Beyond their direct effects, these viruses may shape or respond to the intestinal environment: modulating immune activity, fueling inflammation, and altering epithelial integrity, and thereby they can be speculated to influence CRC development and therapy response.

Bacteriophages add another layer of complexity [181]. They can modulate human physiology, even though the scale of their influence is yet to be known [181]. It has been proposed that these entities can selectively kill bacteria and promote driver–passenger dynamics within the microbiome, further promoting CRC carcinogenesis [182–185]. Through bacteriolysis, phages release bacterial debris, DNA and lipopolysaccharides that act as pathogen-associated molecular patterns (PAMPs), triggering immune responses and inflammation [186]. They can also access epithelial cells [187], promoting the growth of pathobionts that are able to damage intestinal cells and further promote inflammation [188]. Even though it is known that phages can cross the intestinal barrier by transcytosis of epithelial cells [187], the potential effects that phage particles might exert on the cellular environment are yet to be known [186].

Depending on the microenvironment, diet, and host microbiome composition, phages may either constrain or support pathogenic bacteria. Antibiotic use further enhances horizontal gene transfer between bacteria and phages. In a positive light, phages can modulate immune responses by reducing key cytokines [189], as well as kill possible damaging bacterial taxa [190].

Together, these findings highlight that viruses and phages are not only potential independent players in CRC pathogenesis but also key modulators of the gut microenvironment, shaping inflammation, immune responses and gut permeability. They may also have a role in the modulation of microbiome composition.

Interestingly, and as mentioned before, even though viruses seem to represent such profound relevance for CRC initiation, progression, and treatment outcomes, none of the articles included in this review mentions them. Furthermore, even though all of them declare that their objective is to analyze microbiota composition, sequencing methodological procedures are biased in favor of bacteria (with Bellerba et al. only isolating bacterial DNA to realize whole metagenome shotgun sequencing, and the remaining studies performing 16S rRNA sequencing).

Although biased, the methodological choices of these studies are understandable given the technical challenges in virome research. Sampling, processing and data interpretation all face major limitations. Most DNA extraction kits are optimized for bacteria rather than viruses, leading to viral disruption and loss during processing, which in turn produces poor viral detection [191]. Viral genomes are highly diverse, and many gut viruses remain uncharacterized. With incomplete reference databases, metagenomic pipelines often fail to classify or even detect novel viral reads (“viral dark matter”), underestimating the virome’s diversity and its links to CRC [191–193].

This bias is compounded by the lack of standardized protocols: different studies use different extraction kits, sequencing depths and bioinformatic pipelines, making cross-study comparisons difficult [194,195]. Viral DNA and RNA are also far less abundant than bacterial nucleic acids in stool samples, further reducing detection sensitivity [191]. Consequently, low-level viral populations that might influence CRC are often missed. Even when viral nucleic acids are detected, it is still difficult to determine whether they represent active or latent infection, or simply a transient presence [191].

Together, these factors severely limit our ability to establish a causal link between viruses and CRC carcinogenesis. It is also worth noting that the host cellular environment likely shapes viral behavior, influencing their oncogenic potential and interactions with the other constituents within a particular environment.

### 4.8 Tumor-Intrinsic Characteristics

Another axis of heterogeneity is defined by tumor-intrinsic variables, including anatomic location and disease stage. Historically, CRC includes both colon cancer and rectal cancer. However, growing evidence suggests that colon and rectal cancers are biologically and clinically distinct tumor entities [196,197]. Adding to this, tumor sidedness also presents as another layer of heterogeneity, since it presents distinct biological and molecular characteristics, which leads to different therapeutic strategies and response to therapy [197].

Right-sided tumors are enriched in MSI-high and BRAF mutations, frequently display hypermethylation phenotypes, and show stronger immune infiltration [198]. They also harbor higher levels of *F. nucleatum*, linked to both MSI-H and CIMP-H [199,200]. This bacterium is thought to promote a pro-inflammatory microenvironment [151,201], where ROS-induced DNA hypermethylation may silence *MutL homolog 1* (MLH1) [202], leading to MSI [203], impaired apoptosis, and progression from adenoma to carcinoma [153,204]. In contrast, left-sided tumors more commonly display chromosomal instability, and different microbial communities [198]. These molecular and microbial distinctions align with the CMS classification, in which CMS1 tumors are more frequent among right-sided cancers, while CMS2 and CMS3 predominate in left-sided cases.

These site-specific differences in microbiome composition and metabolome [197,205] suggest that tumor location influences the microbiome’s impact on tumor development and progression. Yet, most of the included studies in this review do not take this into account. In fact, only Žukauskaitė et al. restrict their study to left-sided CRC [36]. While Aarnoutse et al., Huang et al., Li et al. and Bellerba et al. studies include multiple locations (right-sided, left-sided, rectum) [37,39–41], Sánchez-Alcoholado et al. do not identify the tumor location of the included patients [38].

Most included studies used stool samples to assess the gut microbiome, which reflects the overall composition of the colon and rectum, but not site-specific profiles [206]. In the context of MPE-microbiology, this characteristic is fundamental to validate possible inferences of causality between bacterial species and pathogenic mechanisms. Evidence suggests fecal and tissue samples capture different microbial communities: fecal samples better represent the intraluminal microbiome and may serve for noninvasive diagnosis or as prognosis biomarkers, whereas tissue samples are more relevant for studying pathophysiological roles in CRC, since it is more likely that this microbiome is the one responsible for directly interacting with colonocytes and modulating tumor microenvironment [207,208]. In practice, fresh-frozen tissue is rarely available, and formalin-fixed paraffin-embedded (FFPE) blocks, though widely used, pose challenges due to microbial contamination and compositional alterations during processing. In the future, microbiome studies in CRC should focus on tumor or mucosal samples, in order to analyze site-specific microbiomes.

Additionally, the composition of the gut microbiota varies with disease progression, with CRC patients at different TNM stages exhibiting distinct microbial profiles. Patients with stage III–IV disease demonstrate significantly higher diversity in their gut microbiome compared to those with stage I–II [209]. Similarly, microbial composition differs between metastatic and non-metastatic CRC cases [210]. While Žukauskaitė et al. do not report tumor staging [36], Aarnoutse et al. likely include only stage IV patients, as the study focuses on mCRC [37]. In contrast, Huang et al., Li et al., and Bellerba et al. include patients with stages I to III [39–41], while Sánchez-Alcoholado et al. include only stages II and III [38]. Altogether, these findings reinforce the notion that CRC is not a uniform disease that can be influenced by tumor location and stage. A more refined, stratified approach in both research and clinical practice is essential for advancing our understanding and treatment of CRC.

### 4.9 Therapeutic Intervention and Microbiome

CRC therapies presented in this review are not homogeneous, each having differing mechanisms of action and intensity.

Besides Aarnoutse et al. [37], every patient underwent surgical resection. As stated previously by Žukauskaitė et al., bowel preparation and colorectal resection may have a significant impact on the composition of the microbiome [36]. Recent evidence highlights the sensitivity of gut microbiota to antibiotics, bowel preparation, and surgery. Even though the mechanisms in which they intervene are poorly understood, there are variables to be considered when discussing gut microbiome modulation in a clinical context.

In Aarnoutse et al., Sánchez-Alcoholado et al. and Huang et al. studies, the treatment regimen includes capecitabine [37–39]. This drug is used in different settings in the treatment of CRC: monotherapy, adjuvant or neoadjuvant treatment or combined with other therapeutic agents [211]. Furthermore, Li et al. and Bellerba et al., while not disclosing exactly what the scheduled treatment precisely included, it could be speculated that it may include this chemotherapeutic drug [40,41].

In addition to the effects of this drug on gut microbiome composition, the other therapeutic agents used in combination also significantly influence the microbiome, which in turn affects both treatment efficacy and adverse effects. As each therapeutic agent may exert its therapeutic effect in distinct mechanisms of action, it could be speculated that each therapeutic agent may also interact and modulate the gut microbiome composition in different ways.

In Huang et al. [39] and Li et al. [40] studies, it introduces direct microbiota-targeting interventions: probiotics and herbal medicine. As explored before, each also has distinct mechanisms of action, further emphasizing how different interventions uniquely modulate gut microbiome composition.

Finally, Bellerba et al. [41] study implemented Vitamin D supplementation to evaluate how it would impact the gut microbiome. In contrast with the previous interventions presented, such as standard CRC treatments and direct microbiota-targeting interventions, vitamin D is a host factor that can indirectly affect microbiome composition, and thereby influencing immune responses, intestinal barrier integrity, and inflammatory processes [65].

The VDR contributes to the maintenance of gut barrier function by stabilizing tight junctions between intestinal epithelial cells and upregulating key junctional proteins, including ZO-1 and occludin [212,213]. Additionally, VDR negatively regulates NF-kB activation, as its absence has been associated with increased inflammation [214].

Vitamin D also exerts important effects on both the innate and adaptive immune systems. While promoting immune homeostasis by reducing Th17 cell activity, it also inhibits DC differentiation, leading to a more tolerogenic state characterized by reduced Th1 cells and enhanced Treg function [215,216]. Although paradoxical, given that Tregs can contribute to an immunosuppressive microenvironment and therefore promote tumor development, they have also been shown to be beneficial in settings with increased pro-inflammatory cells promoting tumor progression [217]. This framework may apply to the Bellerba et al. [41] study, which involved CRC patients post-treatment and without active neoplasia. In this context, the focus is to restore immune balance and reduce inflammation rather than inhibiting tumor growth.

Supporting the beneficial effects of increased vitamin D levels, another study reported that individuals with higher vitamin D levels exhibited greater α-diversity and β-diversity in their gut microbiome and were more likely to possess butyrate-producing bacteria [218]. This suggests that different therapeutic interventions have distinct and potentially opposing effects on gut microbiome composition, highlighting the need to understand specific therapeutic mechanisms on gut microbiome.

It is also interesting to denote that, although different therapies may lead to treatment-related toxicity, the microbial genera involved in mitigating these AE vary, reflecting the distinct mechanisms of action of each therapy. As previously discussed, butyrate has been shown to enhance radiotherapy efficacy while protecting healthy mucosa, thereby reducing treatment-related toxicity [55]. This aligns with the findings of Sánchez-Alcoholado et al. [38], who observed an enrichment of multiple butyrate-producing genera: *Roseburia, Faecalibacterium, Ruminococcus* in the R group. Conversely, Huang et al. [39] reported a reduction in Lactobacillus in the placebo group, which was restored by probiotics. This is relevant since oxaliplatin has been shown to deplete *Lactobacillus* [162]; when restoring Lactobacillus levels, it also decreased chemotherapy-associated toxicity [219], which could explain why probiotic group showed reduced treatment-related AE.

**Figure 4:**
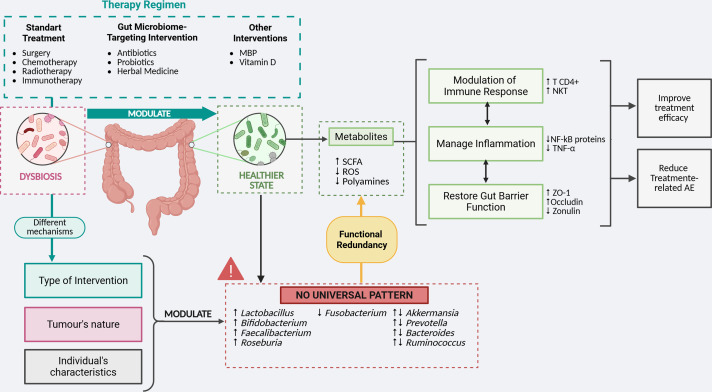
Schematic diagram of the mutual influence between the gut microbiome and therapeutic regimens. CRC patients typically present a dysbiotic microbiome, which is further modulated by therapeutic interventions. These treatments often shift microbiota composition, enriching protective taxa while reducing pro-carcinogenic ones. A healthier microbiome enhances beneficial metabolic activity, notably increasing Short-Chain Fatty Acids (SCFA) and decreasing polyamines and Reactive Oxygen Species (ROS). The byproducts are essential in modulating immune response, managing inflammation, and restoring gut barrier function. Through various pathways, microbial metabolites help reverse the immunosuppressive tumor microenvironment and enhance anti-tumor immunity. They also reduce pro-inflammatory mediators, promoting immune homeostasis. Reduced inflammation, along with increased expression of Tight-Junction Proteins (TJP), further promotes gut barrier function. Together, these changes contribute to improved treatment efficacy and reduced therapy-related Adverse Effects (AE). Despite these consistent clinical outcomes, no universal microbial pattern has been identified. This may reflect functional redundancy within the gut microbiome, where different microorganisms produce similar functional effects. Variations may also arise from differences in therapeutic regimens (in both mechanisms of action and intensity), tumor characteristics (such as location and stage), and individual patient factors: modifiable (diet, lifestyle, metabolic health) and non-modifiable (age, sex). AE, Adverse Effects; CD4^+^, Cluster of Differentiation 4^+^; MBP, Mechanical Bowel Preparation; NF-kB, Nuclear Factor kappa B; SCFA, Short-Chain Fatty Acids; TNF-α, Tumor Necrosis Factor α; ROS, Reactive Oxygen Species; ZO-1, Zonula Occludens-1; ↑, Increased Abundance; ↓, Decreased Abundance. Created in BioRender (https://BioRender.com)

In Fig. 4, we can observe the key findings and conclusions drawn from the studies included in this review.

### 4.10 Limitations of This Review

There are some limitations worth noting.

Firstly, six studies with 361 participants were included, providing a limited evidence base and reducing the overall certainty of findings, as assessed by GRADE. Risk of bias was evident across studies when evaluated with RoB2 and ROBINS-I tools. The Žukauskaitė et al. study [36] showed potential deviations from intended interventions, as only 12 OP group participants were analyzed at POD30. In Li et al. study [40], concerns arose from unclear outcome measurements (since it wasn’t explicit which routine treatment was performed) and lack of a predefined study protocol. Aarnoutse et al. study [37] presented bias from confounding due to large imbalances between the number of individuals in Responders and Non-Responders. Taken together, these limitations, compounded by the small sample size and multiple variables, restrict the generalizability of conclusions to the broader CRC population.

Second, there was substantial heterogeneity across study designs, interventions, and outcomes assessed. Even though this was expected, and in that line was prespecified a narrative synthesis approach, this precludes a quantitative synthesis approach and further limits comparability.

Third, methodological heterogeneity existed in microbiome assessment. Sequencing strategies differed, as did processing pipelines. Normalization procedures and diversity metrics were not standardized, with some studies reporting Shannon or Simpson indices for α-diversity, while others used Bray–Curtis or UniFrac for β-diversity. These discrepancies limit comparability and increase the risk that observed differences reflect analytical choices rather than true biological variation.

Fourth, this review reports different taxonomic levels: genus, species, and strain levels. This was a conscious choice made by the authors in order to identify patterns in microbiome composition and diversity and, therefore, allow cross-study comparisons. However, we recognize that such variation in reporting reduces precision and risks overgeneralization when taxa with very different functional roles are grouped together.

Follow-up periods were distinct between studies, often being short, which can limit the assessment of longitudinal effects, such as microbiome composition alterations, their metabolites, and the manifestation of gastrointestinal toxicity.

Additionally, most studies relied primarily on descriptive taxonomic analyses and did not incorporate metabolomics or functional profiling, restricting the understanding of the influence of microbiome composition, function, and their role in disease processes or therapeutic intervention.

Collectively, these limitations underscore the need for larger, methodologically standardized studies with longer follow-up and integrated multi-omic analyses.

## 5 Conclusion

In conclusion, the studies included in this review reinforce that standard treatment as well as interventions targeting the gut microbiome can meaningfully influence CRC outcomes by modulating gut microbiome constitution, which leads to better immune responses and metabolic activity.

Furthermore, it shows the complexity of CRC lies in its multilayered heterogeneity, with exogenous and endogenous factors affecting the CRC development. The microbiome intersects with these processes, both influencing and being influenced by host and environmental factors. Tumor-intrinsic variables such as location and stage add further complexity, while therapeutic interventions reshape and are reshaped by the microbiome.

The variability in microbiome composition across studies highlights the need for personalized microbiome modulation as part of advancing precision oncology. The MPE framework provides an integrative approach to unravel the pathways that underlie interindividual variability in CRC development and therapy response. Future research should move beyond single-factor approaches, embracing this heterogeneity to design interventions tailored to each patient’s unique context.

## Supplementary Materials





## Data Availability

Not applicable.

## Supplementary Materials

The supplementary material is available online at https://www.techscience.com/doi/10.32604/or.2025.070281/s1.

## Abbreviations

25(OH)D: 25-hydroxyvitamin D
5-FU: Fluorouracil
5-HT: 5-hydroxytryptamine
AE: Adverse effects
AhR: Aryl hydrocarbon receptor
AMPK: AMP-activated protein kinase
aoCRC: Average-onset CRC
BMI: Body mass index
BRAF: Proto-oncogene B-Raf
CD: Cluster of differentiation
CIMP: CpG island methylator phenotype
CIN: Chromosomal instability
CMS: Consensus molecular subtype
CMV: Cytomegalovirus
CRC: Colorectal cancer
CT: Computed tomography
DC: Dendritic cells
DFS: Disease free-survival
DOI: Digital object identifier
EBV: Epstein–Barr virus
EMT: Epithelial-mesenchymal transition
eoCRC: Early-onset CRC
ETBF: Enterotoxigenic *B. fragilis*
FASN: Fatty acid synthase
FFPE: Formalin-fixed paraffin-embedded
GQD: Gegen Qinlian decoction:
GRADE: Grading of recommendations, assessment, development and evaluations
Gy: Gray
HPV: Human papillomavirus
IFN: Interferon
IL: Interleukin
INPLASY: International platform of registered systematic review and meta-analysis protocols
JAK-STAT: Janus kinase/signal transduction and transcription
JCV: John Cunningham virus
KEGG: Kyoto encyclopedia of genes and genomes
KRAS: Kirsten rat sarcoma virus
LPS: Lipopolysaccharide
MBP: Mechanical bowel preparation
MeSH Terms: Medical subject headings terms
MLH1: MutL homolog 1
MPE: Molecular pathological epidemiology
MRI: Magnetic resonance imaging
MSI: Microsatellite instability
N1-AcPUT: N1-acetylputrescine
N1-AcSP: N1-acetylspermine
N1-AcSPD: N1-acetylspermidine
N1, N8-DiAcSPD: N1, N8-diacetylspermidine
N1, N12-DiAcSP: N1, N12-diacetylspermine
N8-AcSPD: N8-acetylspermidine
NF-κB: Nuclear Factor kappa B
NK: Natural Killer
NKT: Natural Killer T
NR: Non-responders
nRCT: Neoadjuvant radiochemotherapy
ODC: Ornithine decarboxylase
OP: Osmotic diarrhea-inducing oral preparation
PAMPs: Pathogen-associated molecular patterns
PD: Progressive disease
PD-1: Programmed cell death protein-1
POD: Postoperative day
PR: Partial response
PRISMA: Preferred reporting items for systematic reviews and meta-analyses
R: Responders
RCTs: Randomized controlled trials
RE: Rectal enema
RECIST: Response evaluation criteria in solid tumors
Robvis: Risk-of-bias visualization
ROS: Reactive oxygen species
SB: Stable disease
SCFA: Short-chain fatty acids
SCNA: Somatic copy number alterations
SoF: Summary of findings
TGF: Transforming growth factor
TGR: Tumor regression grades
TJP: Tight junction proteins
TLR4: Toll-like receptor 4
TNF-α: Tumor necrosis factor α
Treg: T regulatory
VDR: Vitamin D receptor
VEGF: Vascular endothelial growth factor
Wnt/Myc: Wingless-related integration site/myelocytomatosis oncogene
XELOX: Capecitabine and oxaliplatin
ZO-1: Zonula occludens-1

## References

[1] Bray F, Laversanne M, Sung H, Ferlay J, Siegel RL, Soerjomataram I, et al. Global cancer statistics 2022: GLOBOCAN estimates of incidence and mortality worldwide for 36 cancers in 185 countries. CA Cancer J Clin. 2024;74(3):229–63. doi:10.3322/caac.21834; 38572751

[2] Abedizadeh R, Majidi F, Khorasani HR, Abedi H, Sabour D. Colorectal cancer: a comprehensive review of carcinogenesis, diagnosis, and novel strategies for classified treatments. Cancer Metastasis Rev. 2024;43(2):729–53. doi:10.1007/s10555-023-10158-3; 38112903

[3] Matsuda T, Fujimoto A, Igarashi Y. Colorectal cancer: epidemiology, risk factors, and public health strategies. Digestion. 2025;106(2):91–9. doi:10.1159/000543921; 39938491

[4] Siegel RL, Miller KD, Fedewa SA, Ahnen DJ, Meester RGS, Barzi A, et al. Colorectal cancer statistics, 2017. CA Cancer J Clin. 2017;67(3):177–93. doi:10.3322/caac.21395; 28248415

[5] Lythgoe MP, Mullish BH, Frampton AE, Krell J. Polymorphic microbes: a new emerging hallmark of cancer. Trends Microbiol. 2022;30(12):1131–4. doi:10.1016/j.tim.2022.08.004; 36058787

[6] Berg G, Rybakova D, Fischer D, Cernava T, Vergès MC, Charles T, et al. Correction to: microbiome definition re-visited: old concepts and new challenges. Microbiome. 2020;8:119. doi:10.1186/s40168-020-00875-0; 32819450 PMC7441691

[7] Rebersek M. Gut microbiome and its role in colorectal cancer. BMC Cancer. 2021;21(1):1325. doi:10.1186/s12885-021-09054-2; 34895176 PMC8666072

[8] Wong SH, Yu J. Gut microbiota in colorectal cancer: mechanisms of action and clinical applications. Nat Rev Gastroenterol Hepatol. 2019;16(11):690–704. doi:10.1038/s41575-019-0209-8; 31554963

[9] Sedlak JC, Yilmaz ÖH, Roper J. Metabolism and colorectal cancer. Annu Rev Pathol Mech Dis. 2023;18:467–92. doi:10.1146/annurev-pathmechdis-031521-041113; 36323004 PMC9877174

[10] Tjalsma H, Boleij A, Marchesi JR, Dutilh BE. A bacterial driver-passenger model for colorectal cancer: beyond the usual suspects. Nat Rev Microbiol. 2012;10(8):575–82. doi:10.1038/nrmicro2819; 22728587

[11] Louis P, Hold GL, Flint HJ. The gut microbiota, bacterial metabolites and colorectal cancer. Nat Rev Microbiol. 2014;12(10):661–72. doi:10.1038/nrmicro3344; 25198138

[12] Hanus M, Parada-Venegas D, Landskron G, Wielandt AM, Hurtado C, Alvarez K, et al. Immune system, microbiota, and microbial metabolites: the unresolved triad in colorectal cancer microenvironment. Front Immunol. 2021;12:612826. doi:10.3389/fimmu.2021.612826; 33841394 PMC8033001

[13] Hanahan D. Hallmarks of cancer: new dimensions. Cancer Discov. 2022;12(1):31–46. doi:10.1158/2159-8290.cd-21-1059; 35022204

[14] Helmink BA, Wadud Khan MA, Hermann A, Gopalakrishnan V, Wargo JA. The microbiome, cancer, and cancer therapy. Nat Med. 2019;25(3):377–88. doi:10.1038/s41591-019-0377-7; 30842679

[15] Alexander JL, Wilson ID, Teare J, Marchesi JR, Nicholson JK, Kinross JM. Gut microbiota modulation of chemotherapy efficacy and toxicity. Nat Rev Gastroenterol Hepatol. 2017;14(6):356–65. doi:10.1038/nrgastro.2017.20; 28270698

[16] Gaines S, Shao C, Hyman N, Alverdy JC. Gut microbiome influences on anastomotic leak and recurrence rates following colorectal cancer surgery. Br J Surg. 2018;105(2):e131–41. doi:10.1002/bjs.10760; 29341151 PMC5903685

[17] Lauka L, Reitano E, Carra MC, Gaiani F, Gavriilidis P, Brunetti F, et al. Role of the intestinal microbiome in colorectal cancer surgery outcomes. World J Surg Onc. 2019;17:204. doi:10.1186/s12957-019-1754-x; 31791356 PMC6889350

[18] Ralls MW, Miyasaka E, Teitelbaum DH. Intestinal microbial diversity and perioperative complications. J Parenter Enteral Nutr. 2014;38(3):392–9. doi:10.1177/0148607113486482; 23636012 PMC4183124

[19] Bachmann R, Leonard D, Delzenne N, Kartheuser A, Cani PD. Novel insight into the role of microbiota in colorectal surgery. Gut. 2017;66(4):738–49. doi:10.1136/gutjnl-2016-312569; 28153961

[20] Souza DG, Vieira AT, Soares AC, Pinho V, Nicoli JR, Vieira LQ, et al. The essential role of the intestinal microbiota in facilitating acute inflammatory responses. J Immunol. 2004;173(6):4137–46. doi:10.4049/jimmunol.173.6.4137; 15356164

[21] Stavrou G, Kotzampassi K. Gut microbiome, surgical complications and probiotics. Ann Gastroenterol. 2016;30(1):45. doi:10.20524/aog.2016.0086; 28042237 PMC5198246

[22] Aoyama T, Oba K, Honda M, Sadahiro S, Hamada C, Mayanagi S, et al. Impact of postoperative complications on the colorectal cancer survival and recurrence: analyses of pooled individual patients’ data from three large phase III randomized trials. Cancer Med. 2017;6(7):1573–80. doi:10.1002/cam4.1126; 28639738 PMC5504309

[23] Genua F, Raghunathan V, Jenab M, Gallagher WM, Hughes DJ. The role of gut barrier dysfunction and microbiome dysbiosis in colorectal cancer development. Front Oncol. 2021;11:626349. doi:10.3389/fonc.2021.626349; 33937029 PMC8082020

[24] Alasiri GA. Effect of gut microbiota on colorectal cancer progression and treatment. Saudi Med J. 2022;43(12):1289–99. doi:10.15537/smj.2022.43.12.20220367; 36517053 PMC9994512

[25] Liu Y, Lau HC, Yu J. Microbial metabolites in colorectal tumorigenesis and cancer therapy. Gut Microbes. 2023;15(1):2203968. doi:10.1080/19490976.2023.2203968; 37095682 PMC10132243

[26] Qu R, Zhang Y, Ma Y, Zhou X, Sun L, Jiang C, et al. Role of the gut microbiota and its metabolites in tumorigenesis or development of colorectal cancer. Adv Sci. 2023;10(23):2205563. doi:10.1002/advs.202205563; 37263983 PMC10427379

[27] Page MJ, McKenzie JE, Bossuyt PM, Boutron I, Hoffmann TC, Mulrow CD, et al. The PRISMA 2020 statement: an updated guideline for reporting systematic reviews. BMJ. 2021;372:1–9. doi:10.1136/bmj.n71; 33782057 PMC8005924

[28] Saaiq M, Ashraf B. Modifying “Pico” question into “Picos” model for more robust and reproducible presentation of the methodology employed in a scientific study. World J Plast Surg. 2017;6(3):390–2; 29218294 PMC5714990

[29] PICO Portal [Internet]. St. Petersburg, FL, USA: PICO Portal; [cited 2025 Nov 12]. Available from: https://www.picoportal.org.

[30] McGuinness LA, Higgins JPT. Risk-of-bias VISualization (robvis): an R package and Shiny web app for visualizing risk-of-bias assessments. Res Synth Methods. 2021;12(1):55–61. doi:10.1002/jrsm.1411; 32336025

[31] Sterne JAC, Savović J, Page MJ, Elbers RG, Blencowe NS, Boutron I, et al. RoB 2: a revised tool for assessing risk of bias in randomised trials. BMJ. 2019;366:1–58. doi:10.1136/bmj.l4898; 31462531

[32] Sterne J, Higgins J. ROBINS-I V2 Development Group. The risk of bias in non-randomized studies–of interventions, version 2 (ROBINS-I V2) assessment tool [Internet]. [cited 2025 Dec 1]. Available from: https://www.riskofbias.info/welcome/robins-i-v2.

[33] Guyatt GH, Oxman AD, Schünemann HJ, Tugwell P, Knottnerus A. GRADE guidelines: a new series of articles in the journal of clinical epidemiology. J Clin Epidemiol. 2011;64(4):380–2. doi:10.1016/j.jclinepi.2010.09.011; 21185693

[34] Schünemann HJ, Cuello C, Akl EA, Mustafa RA, Meerpohl JJ, Thayer K, et al. GRADE guidelines: 18. How ROBINS-I and other tools to assess risk of bias in nonrandomized studies should be used to rate the certainty of a body of evidence. J Clin Epidemiol. 2019;111:105–14. doi:10.1016/j.jclinepi.2018.01.012; 29432858 PMC6692166

[35] Page M, Higgins J, Sterne J. Chapter 13: assessing risk of bias due to missing evidence in a meta-analysis. In: Cochrane handbook for systematic reviews of interventions. London, UK: Cochrane; 2024. doi:10.1002/9781119536604.ch13.

[36] Žukauskaitė K, Horvath A, Gricius Ž, Kvietkauskas M, Baušys B, Dulskas A, et al. Impact of mechanical bowel preparation on the gut microbiome of patients undergoing left-sided colorectal cancer surgery: randomized clinical trial. Br J Surg. 2024;111(9):1–10. doi:10.1093/bjs/znae213; 39222391 PMC11368128

[37] Aarnoutse R, Ziemons J, de Vos-Geelen J, Valkenburg-Van Iersel L, Wildeboer ACL, Vievermans A, et al. The role of intestinal microbiota in metastatic colorectal cancer patients treated with capecitabine. Clin Colorectal Cancer. 2022;21(2):e87–97. doi:10.1016/j.clcc.2021.10.004; 34801414

[38] Sánchez-Alcoholado L, Laborda-Illanes A, Otero A, Ordóñez R, González-González A, Plaza-Andrades I, et al. Relationships of gut microbiota composition, short-chain fatty acids and polyamines with the pathological response to neoadjuvant radiochemotherapy in colorectal cancer patients. Int J Mol Sci. 2021;22(17):9549. doi:10.3390/ijms22179549; 34502456 PMC8430739

[39] Huang F, Li S, Chen W, Han Y, Yao Y, Yang L, et al. Postoperative probiotics administration attenuates gastrointestinal complications and gut microbiota dysbiosis caused by chemotherapy in colorectal cancer patients. Nutrients. 2023;15(2):356. doi:10.3390/nu15020356; 36678227 PMC9861237

[40] Li Y, Li ZX, Xie CY, Fan J, Lv J, Xu XJ, et al. Gegen Qinlian decoction enhances immunity and protects intestinal barrier function in colorectal cancer patients via gut microbiota. World J Gastroenterol. 2020;26(48):7633–51. doi:10.3748/wjg.v26.i48.7633; 33505141 PMC7789057

[41] Bellerba F, Serrano D, Johansson H, Pozzi C, Segata N, NabiNejad A, et al. Colorectal cancer, vitamin D and microbiota: a double-blind phase II randomized trial (ColoViD) in colorectal cancer patients. Neoplasia. 2022;34:100842. doi:10.1016/j.neo.2022.100842; 36279751 PMC9594107

[42] Jin Y, Liu Y, Zhao L, Zhao F, Feng J, Li S, et al. Gut microbiota in patients after surgical treatment for colorectal cancer. Environ Microbiol. 2019;21(2):772–83. doi:10.1111/1462-2920.14498; 30548192 PMC7379540

[43] Cong J, Zhu H, Liu D, Li T, Zhang C, Zhu J, et al. A pilot study: changes of gut microbiota in post-surgery colorectal cancer patients. Front Microbiol. 2018;9:2777. doi:10.3389/fmicb.2018.02777; 30515141 PMC6255893

[44] Drago L, Toscano M, De Grandi R, Casini V, Pace F. Persisting changes of intestinal microbiota after bowel lavage and colonoscopy. Eur J Gastroenterol Hepatol. 2016;28(5):532–7. doi:10.1097/MEG.0000000000000581; 27015015

[45] Koskenvuo L, Lunkka P, Varpe P, Hyöty M, Satokari R, Haapamäki C, et al. Morbidity after mechanical bowel preparation and oral antibiotics prior to rectal resection: the MOBILE2 randomized clinical trial. JAMA Surg. 2024;159(6):606. doi:10.1001/jamasurg.2024.0184; 38506889 PMC10955353

[46] Peters GJ. Novel developments in the use of antimetabolites. Nucleosides Nucleotides Nucleic Acids. 2014;33(4–6):358–74. doi:10.1080/15257770.2014.894197; 24940694

[47] Saif MW. Targeting cancers in the gastrointestinal tract: role of capecitabine. Onco Targets Ther. 2009;2:29–41. doi:10.2147/ott.s3469; 20616892 PMC2886333

[48] Shin AE, Giancotti FG, Rustgi AK. Metastatic colorectal cancer: mechanisms and emerging therapeutics. Trends Pharmacol Sci. 2023;44(4):222–36. doi:10.1016/j.tips.2023.01.003; 36828759 PMC10365888

[49] Marin JJG, Macias RIR, Monte MJ, Herraez E, Peleteiro-Vigil A, de Blas BS, et al. Cellular mechanisms accounting for the refractoriness of colorectal carcinoma to pharmacological treatment. Cancers. 2020;12(9):2605. doi:10.3390/cancers12092605; 32933095 PMC7563523

[50] Benson AB, Venook AP, Adam M, Chang G, Chen YJ, Ciombor KK, et al. Colon cancer, version 3. 2024, NCCN clinical practice guidelines in oncology. J Natl Compr Cancer Netw. 2024;22(2D):1–26. doi:10.6004/jnccn.2024.0029; 38862008

[51] Chen C, Hou S, Zhao F, Wu B, Liu T, Zhang Z, et al. Application of bevacizumab combined with chemotherapy in patients with colorectal cancer and its effects on brain-gut peptides, intestinal flora, and oxidative stress. Front Surg. 2022;9:872112. doi:10.3389/fsurg.2022.872112; 35478726 PMC9035672

[52] Häfner MF, Debus J. Radiotherapy for colorectal cancer: current standards and future perspectives. Visc Med. 2016;32(3):172–7. doi:10.1159/000446486; 27493944 PMC4945782

[53] George TJ, Franke AJ, Chakravarthy AB, Das P, Dasari A, El-Rayes BF, et al. National Cancer Institute (NCI) state of the science: targeted radiosensitizers in colorectal cancer. Cancer. 2019;125(16):2732–46. doi:10.1002/cncr.32150; 31017664 PMC6663584

[54] Yi Y, Shen L, Shi W, Xia F, Zhang H, Wang Y, et al. Gut microbiome components predict response to neoadjuvant chemoradiotherapy in patients with locally advanced rectal cancer: a prospective, longitudinal study. Clin Cancer Res. 2021;27(5):1329–40. doi:10.1158/1078-0432.ccr-20-3445; 33298472

[55] Park M, Kwon J, Shin H, Moon S, Kim S, Shin U, et al. Butyrate enhances the efficacy of radiotherapy via FOXO3A in colorectal cancer patient-derived organoids. Int J Oncol. 2020;57(6):1307–18. doi:10.3892/ijo.2020.5132; 33173975 PMC7646587

[56] Tesniere A, Schlemmer F, Boige V, Kepp O, Martins I, Ghiringhelli F, et al. Immunogenic death of colon cancer cells treated with oxaliplatin. Oncogene. 2010;29(4):482–91. doi:10.1038/onc.2009.356; 19881547

[57] Allegra CJ, Yothers G, O’Connell MJ, Beart RW, Wozniak TF, Pitot HC, et al. Neoadjuvant 5-FU or capecitabine plus radiation with or without oxaliplatin in rectal cancer patients: a phase III randomized clinical trial. J Natl Cancer Inst. 2015;107(11):djv248. doi:10.1093/jnci/djv248; 26374429 PMC4849360

[58] Okamoto K, Nozawa H, Emoto S, Murono K, Sasaki K, Ishihara S. Adjuvant capecitabine and oxaliplatin for elderly patients with colorectal cancer. Oncology. 2022;100(11):576–82. doi:10.1159/000527012; 36252550

[59] Kenneth MJ, Wu CC, Fang CY, Hsu TK, Lin IC, Huang SW, et al. Exploring the impact of chemotherapy on the emergence of antibiotic resistance in the gut microbiota of colorectal cancer patients. Antibiotics. 2025;14(3):264. doi:10.3390/antibiotics14030264; 40149075 PMC11939702

[60] Valdes AM, Walter J, Segal E, Spector TD. Role of the gut microbiota in nutrition and health. BMJ. 2018;361:1–9. doi:10.1136/bmj.k2179; 29899036 PMC6000740

[61] He Y, Fu L, Li Y, Wang W, Gong M, Zhang J, et al. Gut microbial metabolites facilitate anticancer therapy efficacy by modulating cytotoxic CD8^+^ T cell immunity. Cell Metab. 2021;33(5):988–1000. doi:10.1016/j.cmet.2021.03.002; 33761313

[62] Zuo F, Yin L, Yang X, Wu W, Zhong J, Da M, et al. Gut microbiome associated with chemotherapy-induced diarrhea from the CapeOX regimen as adjuvant chemotherapy in resected stage III colorectal cancer. Gut Pathog. 2019;11(1):18. doi:10.1186/s13099-019-0299-4; 31168325 PMC6489188

[63] Vickers A, Zollman C, Lee R. Herbal medicine. West J Med. 2001;175(2):125. doi:10.1136/EWJM.175.2.125; 11483560 PMC1071505

[64] Plum LA, DeLuca HF. Vitamin D, disease and therapeutic opportunities. Nat Rev Drug Discov. 2010;9(12):941–55. doi:10.1038/nrd3318; 21119732

[65] Sun J. Dietary vitamin D, vitamin D receptor, and microbiome. Curr Opin Clin Nutr Metab Care. 2018;21(6):471–4. doi:10.1097/mco.0000000000000516; 30169457 PMC6168421

[66] Cheng Y, Ling Z, Li L. The intestinal microbiota and colorectal cancer. Front Immunol. 2020;11:615056. doi:10.3389/fimmu.2020.615056; 33329610 PMC7734048

[67] Belloni S, Caruso R, Giacon C, Baroni I, Conte G, Magon A, et al. Microbiome-modifiers for cancer-related fatigue management: a systematic review. Semin Oncol Nurs. 2024;40(2):151619. doi:10.1016/j.soncn.2024.151619; 38503656

[68] Quévrain E, Maubert MA, Michon C, Chain F, Marquant R, Tailhades J, et al. Identification of an anti-inflammatory protein from *Faecalibacterium prausnitzii*, a commensal bacterium deficient in Crohn’s disease. Gut. 2016;65(3):415–25. doi:10.1136/gutjnl-2014-307649; 26045134 PMC5136800

[69] Ranjbar M, Salehi R, Haghjooy Javanmard S, Rafiee L, Faraji H, Jafarpor S, et al. The dysbiosis signature of *Fusobacterium nucleatum* in colorectal cancer-cause or consequences? A systematic review. Cancer Cell Int. 2021;21(1):194. doi:10.1186/s12935-021-01886-z; 33823861 PMC8025348

[70] Dai Z, Zhang J, Wu Q, Chen J, Liu J, Wang L, et al. The role of microbiota in the development of colorectal cancer. Int J Cancer. 2019;145(8):2032–41. doi:10.1002/ijc.32017; 30474116 PMC6899977

[71] Anderson DI, Keskey R, Ackerman MT, Zaborina O, Hyman N, Alverdy JC, et al. *Enterococcus faecalis* is associated with anastomotic leak in patients undergoing colorectal surgery. Surg Infect. 2021;22(10):1047–51. doi:10.1089/sur.2021.147; 34255574 PMC8851212

[72] Gagnière J. Gut microbiota imbalance and colorectal cancer. World J Gastroenterol. 2016;22(2):501. doi:10.3748/wjg.v22.i2.501; 26811603 PMC4716055

[73] Wang T, Cai G, Qiu Y, Fei N, Zhang M, Pang X, et al. Structural segregation of gut microbiota between colorectal cancer patients and healthy volunteers. ISME J. 2012;6(2):320–9. doi:10.1038/ismej.2011.109; 21850056 PMC3260502

[74] Chelakkot C, Choi Y, Kim DK, Park HT, Ghim J, Kwon Y, et al. *Akkermansia muciniphila*-derived extracellular vesicles influence gut permeability through the regulation of tight junctions. Exp Mol Med. 2018;50(2):e450. doi:10.1038/emm.2017.282; 29472701 PMC5903829

[75] Qu S, Zheng Y, Huang Y, Feng Y, Xu K, Zhang W, et al. Excessive consumption of mucin by over-colonized *Akkermansia muciniphila* promotes intestinal barrier damage during malignant intestinal environment. Front Microbiol. 2023;14:1111911. doi:10.3389/fmicb.2023.1111911; 36937258 PMC10018180

[76] Baxter NT, Zackular JP, Chen GY, Schloss PD. Structure of the gut microbiome following colonization with human feces determines colonic tumor burden. Microbiome. 2014;2:20. doi:10.1186/2049-2618-2-20; 24967088 PMC4070349

[77] Wu S, Rhee KJ, Albesiano E, Rabizadeh S, Wu X, Yen HR, et al. A human colonic commensal promotes colon tumorigenesis via activation of T helper type 17 T cell responses. Nat Med. 2009;15(9):1016–22. doi:10.1038/nm.2015; 19701202 PMC3034219

[78] Sears CL, Geis AL, Housseau F. *Bacteroides fragilis* subverts mucosal biology: from symbiont to colon carcinogenesis. J Clin Invest. 2014;124(10):4166–72. doi:10.1172/jci72334; 25105360 PMC4191034

[79] Zafar H, Saier Jr MH. Gut Bacteroides species in health and disease. Gut Microbes. 2021;13(1):1848158. doi:10.1080/19490976.2020.1848158; 33535896 PMC7872030

[80] Uronis JM, Mühlbauer M, Herfarth HH, Rubinas TC, Jones GS, Jobin C. Modulation of the intestinal microbiota alters colitis-associated colorectal cancer susceptibility. PLoS One. 2009;4(6):e6026. doi:10.1371/journal.pone.0006026; 19551144 PMC2696084

[81] Liang Q, Chiu J, Chen Y, Huang Y, Higashimori A, Fang J, et al. Fecal bacteria act as novel biomarkers for noninvasive diagnosis of colorectal cancer. Clin Cancer Res. 2017;23(8):2061–70. doi:10.1158/1078-0432.ccr-16-1599; 27697996

[82] Saus E, Iraola-Guzmán S, Willis JR, Brunet-Vega A, Gabaldón T. Microbiome and colorectal cancer: roles in carcinogenesis and clinical potential. Mol Aspects Med. 2019;69:93–106. doi:10.1016/j.mam.2019.05.001; 31082399 PMC6856719

[83] Flemer B, Lynch DB, Brown JMR, Jeffery IB, Ryan FJ, Claesson MJ, et al. Tumour-associated and non-tumour-associated microbiota in colorectal cancer. Gut. 2017;66(4):633–43. doi:10.1136/gutjnl-2015-309595; 26992426 PMC5529966

[84] Zhu Q, Jin Z, Wu W, Gao R, Guo B, Gao Z, et al. Analysis of the intestinal lumen microbiota in an animal model of colorectal cancer. PLoS One. 2014;9(3):e90849. doi:10.1371/journal.pone.0090849; 24603888 PMC3946251

[85] Weir TL, Manter DK, Sheflin AM, Barnett BA, Heuberger AL, Ryan EP. Stool microbiome and metabolome differences between colorectal cancer patients and healthy adults. PLoS One. 2013;8(8):e70803. doi:10.1371/journal.pone.0070803; 23940645 PMC3735522

[86] Zhang X, Yu D, Wu D, Gao X, Shao F, Zhao M, et al. Tissue-resident Lachnospiraceae family bacteria protect against colorectal carcinogenesis by promoting tumor immune surveillance. Cell Host Microbe. 2023;31(3):418–32. doi:10.1016/j.chom.2023.01.013; 36893736

[87] Ze X, Duncan SH, Louis P, Flint HJ. *Ruminococcus bromii* is a keystone species for the degradation of resistant starch in the human colon. ISME J. 2012;6(8):1535–43. doi:10.1038/ismej.2012.4; 22343308 PMC3400402

[88] Thursby E, Juge N. Introduction to the human gut microbiota. Biochem J. 2017;474(11):1823–36. doi:10.1042/bcj20160510; 28512250 PMC5433529

[89] O’Keefe SJD, Ou J, Aufreiter S, O’Connor D, Sharma S, Sepulveda J, et al. Products of the colonic microbiota mediate the effects of diet on colon cancer risk. J Nutr. 2009;139(11):2044–8. doi:10.3945/jn.109.104380; 19741203 PMC6459055

[90] Kim J, Lee HK. Potential role of the gut microbiome in colorectal cancer progression. Front Immunol. 2022;12:807648. doi:10.3389/fimmu.2021.807648; 35069592 PMC8777015

[91] Wang HB, Wang PY, Wang X, Wan YL, Liu YC. Butyrate enhances intestinal epithelial barrier function via up-regulation of tight junction protein Claudin-1 transcription. Dig Dis Sci. 2012;57(12):3126–35. doi:10.1007/s10620-012-2259-4; 22684624

[92] Manco M, Putignani L, Bottazzo GF. Gut microbiota, lipopolysaccharides, and innate immunity in the pathogenesis of obesity and cardiovascular risk. Endocr Rev. 2010;31(6):817–44. doi:10.1210/er.2009-0030; 20592272

[93] Rios-Covian D, Salazar N, Gueimonde M, de Los Reyes-Gavilan CG. Shaping the metabolism of intestinal *Bacteroides* population through diet to improve human health. Front Microbiol. 2017;8:376. doi:10.3389/fmicb.2017.00376; 28326076 PMC5339271

[94] Chen T, Long W, Zhang C, Liu S, Zhao L, Hamaker BR. Fiber-utilizing capacity varies in *Prevotella*-versus *Bacteroides*-dominated gut microbiota. Sci Rep. 2017;7(1):2594. doi:10.1038/s41598-017-02995-4; 28572676 PMC5453967

[95] Reunanen J, Kainulainen V, Huuskonen L, Ottman N, Belzer C, Huhtinen H, et al. *Akkermansia muciniphila* adheres to enterocytes and strengthens the integrity of the epithelial cell layer. Appl Environ Microbiol. 2015;81(11):3655–62. doi:10.1128/aem.04050-14; 25795669 PMC4421065

[96] Derrien M, Vaughan EE, Plugge CM, de Vos WM. *Akkermansia muciniphila* gen. nov., sp. nov., a human intestinal mucin-degrading bacterium. Int J Syst Evol Microbiol. 2004;54(5):1469–76. doi:10.1099/ijs.0.02873-0; 15388697

[97] Chen C, Fang S, Wei H, He M, Fu H, Xiong X, et al. *Prevotella copri* increases fat accumulation in pigs fed with formula diets. Microbiome. 2021;9(1):175. doi:10.1186/s40168-021-01110-0; 34419147 PMC8380364

[98] Fong W, Li Q, Yu J. Gut microbiota modulation: a novel strategy for prevention and treatment of colorectal cancer. Oncogene. 2020;39(26):4925–43. doi:10.1038/s41388-020-1341-1; 32514151 PMC7314664

[99] Zaher K, Basingab F. Interaction between gut microbiota and dendritic cells in colorectal cancer. Biomedicines. 2023;11(12):3196. doi:10.3390/biomedicines11123196; 38137417 PMC10741039

[100] Hurtado CG, Wan F, Housseau F, Sears CL. Roles for interleukin 17 and adaptive immunity in pathogenesis of colorectal cancer. Gastroenterology. 2018;155(6):1706–15. doi:10.1053/j.gastro.2018.08.056; 30218667 PMC6441974

[101] Wei P, Hu GH, Kang HY, Yao HB, Kou W, Liu H, et al. An aryl hydrocarbon receptor ligand acts on dendritic cells and T cells to suppress the Th17 response in allergic rhinitis patients. Lab Investig. 2014;94(5):528–35. doi:10.1038/labinvest.2014.8; 24514067

[102] Korecka A, Dona A, Lahiri S, Tett AJ, Al-Asmakh M, Braniste V, et al. Bidirectional communication between the Aryl hydrocarbon Receptor (AhR) and the microbiome tunes host metabolism. NPJ Biofilms Microbiomes. 2016;2:16014. doi:10.1038/npjbiofilms.2016.14; 28721249 PMC5515264

[103] Mohseni AH, Taghinezhad-S S, Casolaro V, Lv Z, Li D. Potential links between the microbiota and T cell immunity determine the tumor cell fate. Cell Death Dis. 2023;14(2):154. doi:10.1038/s41419-023-05560-2; 36828830 PMC9958015

[104] Van Kaer L, Parekh VV, Wu L. Invariant natural killer T cells: bridging innate and adaptive immunity. Cell Tissue Res. 2011;343(1):43–55. doi:10.1007/s00441-010-1023-3; 20734065 PMC3616393

[105] Burks J, Olkhanud PB, Berzofsky JA. The role of NKT cells in gastrointestinal cancers. OncoImmunology. 2022;11(1):2009666. doi:10.1080/2162402X.2021.2009666; 36524208 PMC9746626

[106] Ihara F, Sakurai D, Takami M, Kamata T, Kunii N, Yamasaki K, et al. Regulatory T cells induce CD4^−^ NKT cell anergy and suppress NKT cell cytotoxic function. Cancer Immunol Immunother. 2019;68(12):1935–47. doi:10.1007/s00262-019-02417-6; 31641795 PMC11028189

[107] Li T, Fu B, Zhang X, Zhou Y, Yang M, Cao M, et al. Overproduction of gastrointestinal 5-HT promotes colitis-associated colorectal cancer progression via enhancing NLRP3 inflammasome activation. Cancer Immunol Res. 2021;9(9):1008–23. doi:10.1158/2326-6066.cir-20-1043; 34285037

[108] Kannen V, Bader M, Sakita JY, Uyemura SA, Squire JA. The dual role of serotonin in colorectal cancer. Trends Endocrinol Metab. 2020;31(8):611–25. doi:10.1016/j.tem.2020.04.008; 32439105

[109] Reigstad CS, Salmonson CE, Rainey JF, Szurszewski JH, Linden DR, Sonnenburg JL, et al. Gut microbes promote colonic serotonin production through an effect of short-chain fatty acids on enterochromaffin cells. FASEB J. 2015;29(4):1395–403. doi:10.1096/fj.14-259598; 25550456 PMC4396604

[110] Bardaweel SK, Gul M, Alzweiri M, Ishaqat A, ALSalamat HA, Bashatwah RM. Reactive oxygen species: the dual role in physiological and pathological conditions of the human body. Eurasian J Med. 2018;50(3):193–201. doi:10.5152/eurasianjmed.2018.17397; 30515042 PMC6263229

[111] Evans MD, Dizdaroglu M, Cooke MS. Oxidative DNA damage and disease: induction, repair and significance. Mutat Res/Rev Mutat Res. 2004;567(1):1–61. doi:10.1016/j.mrrev.2003.11.001; 15341901

[112] Neis E, Dejong C, Rensen S. The role of microbial amino acid metabolism in host metabolism. Nutrients. 2015;7(4):2930–46. doi:10.3390/nu7042930; 25894657 PMC4425181

[113] Chen J, Rao JN, Zou T, Liu L, Marasa BS, Xiao L, et al. Polyamines are required for expression of Toll-like receptor 2 modulating intestinal epithelial barrier integrity. Am J Physiol Gastrointest Liver Physiol. 2007;293(3):1–35. doi:10.1152/ajpgi.00201.2007; 17600044

[114] Hardbower DM, Asim M, Luis PB, Singh K, Barry DP, Yang C, et al. Ornithine decarboxylase regulates M1 macrophage activation and mucosal inflammation via histone modifications. Proc Natl Acad Sci USA. 2017;114(5):1–10. doi:10.1073/pnas.1614958114; 28096401 PMC5293075

[115] Sánchez-Jiménez F, Medina MÁ, Villalobos-Rueda L, Urdiales JL. Polyamines in mammalian pathophysiology. Cell Mol Life Sci. 2019;76(20):3987–4008. doi:10.1007/s00018-019-03196-0; 31227845 PMC11105599

[116] Johnson CH, Dejea CM, Edler D, Hoang LT, Santidrian AF, Felding BH, et al. Metabolism links bacterial biofilms and colon carcinogenesis. Cell Metab. 2015;21(6):891–7. doi:10.1016/j.cmet.2015.04.011; 25959674 PMC4456201

[117] Goodwin AC, Shields CED, Wu S, Huso DL, Wu X, Murray-Stewart TR, et al. Polyamine catabolism contributes to enterotoxigenic *Bacteroides fragilis*-induced colon tumorigenesis. Proc Natl Acad Sci USA. 2011;108(37):15354–9. doi:10.1073/pnas.1010203108; 21876161 PMC3174648

[118] Chagneau CV, Garcie C, Bossuet-Greif N, Tronnet S, Brachmann AO, Piel J, et al. The polyamine spermidine modulates the production of the bacterial genotoxin colibactin. mSphere. 2019;4(5):1–11. doi:10.1128/msphere.00414-19; 31578245 PMC6796968

[119] Holbert CE, Cullen MT, Casero RA Jr, Stewart TM. Polyamines in cancer: integrating organismal metabolism and antitumour immunity. Nat Rev Cancer. 2022;22(8):467–80. doi:10.1038/s41568-022-00473-2; 35477776 PMC9339478

[120] Hayes CS, Shicora AC, Keough MP, Snook AE, Burns MR, Gilmour SK. Polyamine-blocking therapy reverses immunosuppression in the tumor microenvironment. Cancer Immunol Res. 2014;2(3):274–85. doi:10.1158/2326-6066.cir-13-0120-t; 24778323 PMC4101915

[121] Levy M, Thaiss CA, Zeevi D, Dohnalová L, Zilberman-Schapira G, Ali Mahdi J, et al. Microbiota-modulated metabolites shape the intestinal microenvironment by regulating NLRP6 inflammasome signaling. Cell. 2015;163(6):1428–43. doi:10.1016/j.cell.2015.10.048; 26638072 PMC5665753

[122] Davila AM, Blachier F, Gotteland M, Andriamihaja M, Benetti PH, Sanz Y, et al. Intestinal luminal nitrogen metabolism: role of the gut microbiota and consequences for the host. Pharmacol Res. 2013;68(1):95–107. doi:10.1016/j.phrs.2012.11.005; 23183532

[123] Chen W, Liu F, Ling Z, Tong X, Xiang C. Human intestinal lumen and mucosa-associated microbiota in patients with colorectal cancer. PLoS One. 2012;7(6):e39743. doi:10.1371/journal.pone.0039743; 22761885 PMC3386193

[124] Moya A, Ferrer M. Functional redundancy-induced stability of gut microbiota subjected to disturbance. Trends Microbiol. 2016;24(5):402–13. doi:10.1016/j.tim.2016.02.002; 26996765

[125] Li J, Ma X, Chakravarti D, Shalapour S, DePinho RA. Genetic and biological hallmarks of colorectal cancer. Genes Dev. 2021;35(11–12):787–820. doi:10.1101/gad.348226.120; 34074695 PMC8168558

[126] Ionescu VA, Gheorghe G, Bacalbasa N, Chiotoroiu AL, Diaconu C. Colorectal cancer: from risk factors to oncogenesis. Medicina. 2023;59(9):1646. doi:10.3390/medicina59091646; 37763765 PMC10537191

[127] Simon K. Colorectal cancer development and advances in screening. Clin Interv Aging. 2016;11:967–76. doi:10.2147/CIA.S109285; 27486317 PMC4958365

[128] Morgillo F, Dallio M, Della Corte CM, Gravina AG, Viscardi G, Loguercio C, et al. Carcinogenesis as a result of multiple inflammatory and oxidative hits: a comprehensive review from tumor microenvironment to gut microbiota. Neoplasia. 2018;20(7):721–33. doi:10.1016/j.neo.2018.05.002; 29859426 PMC6014569

[129] Ogino S, Lochhead P, Chan AT, Nishihara R, Cho E, Wolpin BM, et al. Molecular pathological epidemiology of epigenetics: emerging integrative science to analyze environment, host, and disease. Mod Pathol. 2013;26(4):465–84. doi:10.1038/modpathol.2012.214; 23307060 PMC3637979

[130] Hamada T, Keum N, Nishihara R, Ogino S. Molecular pathological epidemiology: new developing frontiers of big data science to study etiologies and pathogenesis. J Gastroenterol. 2017;52(3):265–75. doi:10.1007/s00535-016-1272-3; 27738762 PMC5325774

[131] Ogino S, Chan AT, Fuchs CS, Giovannucci E. Molecular pathological epidemiology of colorectal neoplasia: an emerging transdisciplinary and interdisciplinary field. Gut. 2011;60(3):397–411. doi:10.1136/gut.2010.217182; 21036793 PMC3040598

[132] Nishi A, Milner DA Jr, Giovannucci EL, Nishihara R, Tan AS, Kawachi I, et al. Integration of molecular pathology, epidemiology and social science for global precision medicine. Expert Rev Mol Diagn. 2016;16(1):11–23. doi:10.1586/14737159.2016.1115346; 26636627 PMC4713314

[133] Nishihara R, VanderWeele TJ, Shibuya K, Mittleman MA, Wang M, Field AE, et al. Molecular pathological epidemiology gives clues to paradoxical findings. Eur J Epidemiol. 2015;30(10):1129–35. doi:10.1007/s10654-015-0088-4; 26445996 PMC4639412

[134] Nyström M, Mutanen M. Diet and epigenetics in colon cancer. World J Gastroenterol. 2009;15(3):257. doi:10.3748/wjg.15.257; 19140224 PMC2653321

[135] Samowitz WS, Albertsen H, Sweeney C, Herrick J, Caan BJ, Anderson KE, et al. Association of smoking, CpG island methylator phenotype, and V600E BRAF mutations in colon cancer. J Natl Cancer Inst. 2006;98(23):1731–8. doi:10.1093/jnci/djj468; 17148775

[136] Ogino S, Nishihara R, VanderWeele TJ, Wang M, Nishi A, Lochhead P, et al. Review article: the role of molecular pathological epidemiology in the study of neoplastic and non-neoplastic diseases in the era of precision medicine. Epidemiology. 2016;27(4):602–11. doi:10.1097/ede.0000000000000471; 26928707 PMC4892980

[137] Wang M, Kuchiba A, Ogino S. A meta-regression method for studying etiological heterogeneity across disease subtypes classified by multiple biomarkers. Am J Epidemiol. 2015;182(3):263–70. doi:10.1093/aje/kwv040; 26116215 PMC4517696

[138] Rejali L, Seifollahi Asl R, Sanjabi F, Fatemi N, Asadzadeh Aghdaei H, Saeedi Niasar M, et al. Principles of molecular utility for CMS classification in colorectal cancer management. Cancers. 2023;15(10):2746. doi:10.3390/cancers15102746; 37345083 PMC10216373

[139] Guinney J, Dienstmann R, Wang X, de Reyniès A, Schlicker A, Soneson C, et al. The consensus molecular subtypes of colorectal cancer. Nat Med. 2015;21(11):1350–6. doi:10.1038/nm.3967; 26457759 PMC4636487

[140] La Vecchia S, Sebastián C. Metabolic pathways regulating colorectal cancer initiation and progression. Semin Cell Dev Biol. 2020;98:63–70. doi:10.1016/j.semcdb.2019.05.018; 31129171

[141] Murakami T, Akazawa Y, Yatagai N, Hiromoto T, Sasahara N, Saito T, et al. Molecular characterization of sessile serrated adenoma/polyps with dysplasia/carcinoma based on immunohistochemistry, next-generation sequencing, and microsatellite instability testing: a case series study. Diagn Pathol. 2018;13(1):88. doi:10.1186/s13000-018-0771-3; 30458818 PMC6247685

[142] Goel A, Nagasaka T, Arnold CN, Inoue T, Hamilton C, Niedzwiecki D, et al. The CpG island methylator phenotype and chromosomal instability are inversely correlated in sporadic colorectal cancer. Gastroenterology. 2007;132(1):127–38. doi:10.1053/j.gastro.2006.09.018; 17087942

[143] Samowitz WS, Albertsen H, Herrick J, Levin TR, Sweeney C, Murtaugh MA, et al. Evaluation of a large, population-based sample supports a CpG island methylator phenotype in colon cancer. Gastroenterology. 2005;129(3):837–45. doi:10.1053/j.gastro.2005.06.020; 16143123

[144] Nosho K, Irahara N, Shima K, Kure S, Kirkner GJ, Schernhammer ES, et al. Comprehensive biostatistical analysis of CpG island methylator phenotype in colorectal cancer using a large population-based sample. PLoS One. 2008;3(11):e3698. doi:10.1371/journal.pone.0003698; 19002263 PMC2579485

[145] Sanchez JA, Krumroy L, Plummer S, Aung P, Merkulova A, Skacel M, et al. Genetic and epigenetic classifications define clinical phenotypes and determine patient outcomes in colorectal cancer. Br J Surg. 2009;96(10):1196–204. doi:10.1002/bjs.6683; 19787768

[146] Gunter MJ, Leitzmann MF. Obesity and colorectal cancer: epidemiology, mechanisms and candidate genes. J Nutr Biochem. 2006;17(3):145–56. doi:10.1016/j.jnutbio.2005.06.011; 16426829

[147] An H, Jang Y, Choi J, Hur J, Kim S, Kwon Y. New insights into AMPK, as a potential therapeutic target in metabolic dysfunction-associated steatotic liver disease and hepatic fibrosis. Biomol Ther. 2025;33(1):18–38. doi:10.4062/biomolther.2024.188; 39702310 PMC11704404

[148] Ogino S, Nosho K, Meyerhardt JA, Kirkner GJ, Chan AT, Kawasaki T, et al. Cohort study of fatty acid synthase expression and patient survival in colon cancer. J Clin Oncol. 2008;26(35):5713–20. doi:10.1200/jco.2008.18.2675; 18955444 PMC2630484

[149] Permain J, Hock B, Eglinton T, Purcell R. Functional links between the microbiome and the molecular pathways of colorectal carcinogenesis. Cancer Metastasis Rev. 2024;43(4):1463–74. doi:10.1007/s10555-024-10215-5; 39340753 PMC11554747

[150] Purcell RV, Visnovska M, Biggs PJ, Schmeier S, Frizelle FA. Distinct gut microbiome patterns associate with consensus molecular subtypes of colorectal cancer. Sci Rep. 2017;7(1):11590. doi:10.1038/s41598-017-11237-6; 28912574 PMC5599497

[151] Kostic AD, Chun E, Robertson L, Glickman JN, Gallini CA, Michaud M, et al. *Fusobacterium nucleatum* potentiates intestinal tumorigenesis and modulates the tumor-immune microenvironment. Cell Host Microbe. 2013;14(2):207–15. doi:10.1016/j.chom.2013.07.007; 23954159 PMC3772512

[152] Sulit AK, Daigneault M, Allen-Vercoe E, Silander OK, Hock B, McKenzie J, et al. Bacterial lipopolysaccharide modulates immune response in the colorectal tumor microenvironment. NPJ Biofilms Microbiomes. 2023;9:59. doi:10.1038/s41522-023-00429-w; 37612266 PMC10447454

[153] Flanagan L, Schmid J, Ebert M, Soucek P, Kunicka T, Liska V, et al. *Fusobacterium nucleatum* associates with stages of colorectal neoplasia development, colorectal cancer and disease outcome. Eur J Clin Microbiol Infect Dis. 2014;33(8):1381–90. doi:10.1007/s10096-014-2081-3; 24599709

[154] Ternes D, Tsenkova M, Pozdeev VI, Meyers M, Koncina E, Atatri S, et al. The gut microbial metabolite formate exacerbates colorectal cancer progression. Nat Metab. 2022;4(4):458–75. doi:10.1038/s42255-022-00558-0; 35437333 PMC9046088

[155] Larigot L, Juricek L, Dairou J, Coumoul X. AhR signaling pathways and regulatory functions. Biochim Open. 2018;7:1–9. doi:10.1016/j.biopen.2018.05.001; 30003042 PMC6039966

[156] Li J, Huang L, Zhao H, Yan Y, Lu J. The role of interleukins in colorectal cancer. Int J Biol Sci. 2020;16(13):2323–39. doi:10.7150/ijbs.46651; 32760201 PMC7378639

[157] Zhao H, Ming T, Tang S, Ren S, Yang H, Liu M, et al. Wnt signaling in colorectal cancer: pathogenic role and therapeutic target. Mol Cancer. 2022;21(1):144. doi:10.1186/s12943-022-01616-7; 35836256 PMC9281132

[158] Lee CG, Hwang S, Gwon SY, Park C, Jo M, Hong JE, et al. *Bacteroides fragilis* toxin induces intestinal epithelial cell secretion of interleukin-8 by the E-cadherin/β-catenin/NF-κB dependent pathway. Biomedicines. 2022;10(4):827. doi:10.3390/biomedicines10040827; 35453577 PMC9032310

[159] Schatoff EM, Leach BI, Dow LE. WNT signaling and colorectal cancer. Curr Colorectal Cancer Rep. 2017;13(2):101–10. doi:10.1007/s11888-017-0354-9; 28413363 PMC5391049

[160] Li Y, Jia X, Li C, Sun H, Nie S, Giovannucci EL, et al. The global incident gastrointestinal cancers attributable to suboptimal diets from 1990 to 2018. Gastroenterology. 2024;167(6):1141–51. doi:10.1053/j.gastro.2024.07.009; 39019406

[161] Soldán M, Argalášová L, Hadvinová L, Galileo B, Babjaková J. The effect of dietary types on gut microbiota composition and development of non-communicable diseases: a narrative review. Nutrients. 2024;16(18):3134. doi:10.3390/nu16183134; 39339734 PMC11434870

[162] Liu Y, Baba Y, Ishimoto T, Gu X, Zhang J, Nomoto D, et al. Gut microbiome in gastrointestinal cancer: a friend or foe? Int J Biol Sci. 2022;18(10):4101–17. doi:10.7150/ijbs.69331; 35844804 PMC9274484

[163] Hoang T, Kim M, Park JW, Jeong SY, Lee J, Shin A. Dysbiotic microbiome variation in colorectal cancer patients is linked to lifestyles and metabolic diseases. BMC Microbiol. 2023;23(1):33. doi:10.1186/s12866-023-02771-7; 36709262 PMC9883847

[164] Zheng R, Du M, Zhang B, Xin J, Chu H, Ni M, et al. Body mass index (BMI) trajectories and risk of colorectal cancer in the PLCO cohort. Br J Cancer. 2018;119(1):130–2. doi:10.1038/s41416-018-0121-y; 29872147 PMC6035226

[165] Barot SV, Sangwan N, Nair KG, Schmit SL, Xiang S, Kamath S, et al. Distinct intratumoral microbiome of young-onset and average-onset colorectal cancer. eBioMedicine. 2024;100:104980. doi:10.1016/j.ebiom.2024.104980; 38306898 PMC10850116

[166] Wang L, Tu YX, Chen L, Zhang Y, Pan XL, Yang SQ, et al. Male-biased gut microbiome and metabolites aggravate colorectal cancer development. Adv Sci. 2023;10(25):2206238. doi:10.1002/advs.202206238; 37400423 PMC10477899

[167] Selgrad M, Malfertheiner P, Fini L, Goel A, Boland CR, Ricciardiello L. The role of viral and bacterial pathogens in gastrointestinal cancer. J Cell Physiol. 2008;216(2):378–88. doi:10.1002/jcp.21427; 18338378 PMC2855192

[168] Costa NR, Gil da Costa RM, Medeiros R. A viral map of gastrointestinal cancers. Life Sci. 2018;199:188–200. doi:10.1016/j.lfs.2018.02.025; 29476768

[169] Mirzaei H, Goudarzi H, Eslami G, Faghihloo E. Role of viruses in gastrointestinal cancer. J Cell Physiol. 2018;233(5):4000–14. doi:10.1002/jcp.26194; 28926109

[170] Luo Y, Liu Y, Wang C, Gan R. Signaling pathways of EBV-induced oncogenesis. Cancer Cell Int. 2021;21(1):93. doi:10.1186/s12935-021-01793-3; 33549103 PMC7868022

[171] Delecluse S, Tsai MH, Shumilov A, Bencun M, Arrow S, Beshirova A, et al. Epstein-Barr virus induces expression of the LPAM-1 integrin in B cells *in vitro* and *in vivo*. J Virol. 2019;93(5):e01618–18. doi:10.1128/jvi.01618-18; 30541846 PMC6384065

[172] Mahmoudvand S, Shokri S, Nakhaie M, Jalilian FA, Mehri-Ghahfarrokhi A, Yarani R, et al. Small extracellular vesicles as key players in cancer development caused by human oncogenic viruses. Infect Agents Cancer. 2022;17:58. doi:10.1186/s13027-022-00471-x; 36437456 PMC9703759

[173] Burnett-Hartman AN, Newcomb PA, Potter JD. Infectious agents and colorectal cancer: a review of *Helicobacter pylori*, *Streptococcus bovis*, JC virus, and human papillomavirus. Cancer Epidemiol Biomarkers Prev. 2008;17(11):2970–9. doi:10.1158/1055-9965.EPI-08-0571; 18990738 PMC2676114

[174] Bello JOM, Nieva LO, Paredes AC, Gonzalez AMF, Zavaleta LR, Lizano M. Regulation of the Wnt/β-catenin signaling pathway by human papillomavirus E6 and E7 oncoproteins. Viruses. 2015;7(8):4734–55. doi:10.3390/v7082842; 26295406 PMC4576203

[175] Arizmendi-Izazaga A, Navarro-Tito N, Jiménez-Wences H, Mendoza-Catalán MA, Martínez-Carrillo DN, Zacapala-Gómez AE, et al. Metabolic reprogramming in cancer: role of HPV 16 variants. Pathogens. 2021;10(3):347. doi:10.3390/pathogens10030347; 33809480 PMC7999907

[176] Galeone C, Pelucchi C, Vecchia CL. Added sugar, glycemic index and load in colon cancer risk. Curr Opin Clin Nutr Metab Care. 2012;15(4):368–73. doi:10.1097/mco.0b013e3283539f81; 22510682

[177] Sieri S, Krogh V, Agnoli C, Ricceri F, Palli D, Masala G, et al. Dietary glycemic index and glycemic load and risk of colorectal cancer: results from the EPIC-Italy study. Int J Cancer. 2015;136(12):2923–31. doi:10.1002/ijc.29341; 25403784

[178] Mazurek S, Zwerschke W, Jansen-Dürr P, Eigenbrodt E. Effects of the human *Papilloma* virus HPV-16 E7 oncoprotein on glycolysis and glutaminolysis: role of pyruvate kinase type M2 and the glycolytic-enzyme complex. Biochem J. 2001;356(1):247–56. doi:10.1042/0264-6021:; 11336658 PMC1221834

[179] Revello MG, Gerna G. Human cytomegalovirus tropism for endothelial/epithelial cells: scientific background and clinical implications. Rev Med Virol. 2010;20(3):136–55. doi:10.1002/rmv.645; 20084641

[180] Ishaque N, Abba ML, Hauser C, Patil N, Paramasivam N, Huebschmann D, et al. Whole genome sequencing puts forward hypotheses on metastasis evolution and therapy in colorectal cancer. Nat Commun. 2018;9:4782. doi:10.1038/s41467-018-07041-z; 30429477 PMC6235880

[181] Federici S, Nobs SP, Elinav E. Phages and their potential to modulate the microbiome and immunity. Cell Mol Immunol. 2021;18(4):889–904. doi:10.1038/s41423-020-00532-4; 32901128 PMC8115240

[182] Emlet C, Ruffin M, Lamendella R. Enteric virome and carcinogenesis in the gut. Dig Dis Sci. 2020;65(3):852–64. doi:10.1007/s10620-020-06126-4; 32060814

[183] Marônek M, Link R, Monteleone G, Gardlík R, Stolfi C. Viruses in cancers of the digestive system: active contributors or idle bystanders? Int J Mol Sci. 2020;21(21):8133. doi:10.3390/ijms21218133; 33143318 PMC7663754

[184] Hannigan GD, Duhaime MB, Ruffin IVMT, Koumpouras CC, Schloss PD. Diagnostic potential and interactive dynamics of the colorectal cancer virome. mBio. 2018;9(6):e02248–18. doi:10.1128/mbio.02248-18; 30459201 PMC6247079

[185] Khan S, Imran A, Malik A, Ahmad Chaudhary A, Rub A, Jan AT, et al. Bacterial imbalance and gut pathologies: association and contribution of E. coli in inflammatory bowel disease. Crit Rev Clin Lab Sci. 2019;56(1):1–17. doi:10.1080/10408363.2018.1517144; 30373492

[186] Tetz G, Tetz V. Bacteriophages as new human viral pathogens. Microorganisms. 2018;6(2):54. doi:10.3390/microorganisms6020054; 29914145 PMC6027513

[187] Nguyen S, Baker K, Padman BS, Patwa R, Dunstan RA, Weston TA, et al. Bacteriophage transcytosis provides a mechanism to cross epithelial cell layers. mBio. 2017;8(6):e01874–17. doi:10.1128/mbio.01874-17; 29162715 PMC5698557

[188] Handley SA, Devkota S. Going viral: a novel role for bacteriophage in colorectal cancer. mBio. 2019;10(1):e02626–18. doi:10.1128/mBio.02626-18; 30670618 PMC6343040

[189] Budynek P, Dąbrowska K, Skaradziński G, Górski A. Bacteriophages and cancer. Arch Microbiol. 2010;192(5):315–20. doi:10.1007/s00203-010-0559-7; 20232198

[190] Barr JJ, Youle M, Rohwer F. Innate and acquired bacteriophage-mediated immunity. Bacteriophage. 2013;3(3):e25857. doi:10.4161/bact.25857; 24228227 PMC3821666

[191] Garmaeva S, Sinha T, Kurilshikov A, Fu J, Wijmenga C, Zhernakova A. Studying the gut virome in the metagenomic era: challenges and perspectives. BMC Biol. 2019;17(1):84. doi:10.1186/s12915-019-0704-y; 31660953 PMC6819614

[192] Krishnamurthy SR, Wang D. Origins and challenges of viral dark matter. Virus Res. 2017;239:136–42. doi:10.1016/j.virusres.2017.02.002; 28192164

[193] Massimino L, Lovisa S, Antonio Lamparelli L, Danese S, Ungaro F. Gut eukaryotic virome in colorectal carcinogenesis: is that a trigger? Comput Struct Biotechnol J. 2020;19:16–28. doi:10.1016/j.csbj.2020.11.055; 33363706 PMC7750180

[194] Pargin E, Roach MJ, Skye A, Papudeshi B, Inglis LK, Mallawaarachchi V, et al. The human gut virome: composition, colonization, interactions, and impacts on human health. Front Microbiol. 2023;14:963173. doi:10.3389/fmicb.2023.963173; 37293229 PMC10244655

[195] Xu Z, Yeoh YK, Tun HM, Fei N, Zhang J, Morrison M, et al. Variation in the metagenomic analysis of fecal microbiome composition calls for a standardized operating approach. Microbiol Spectr. 2024;12(12):e01516–24. doi:10.1128/spectrum.01516-24; 39475247 PMC11619352

[196] Paschke S, Jafarov S, Staib L, Kreuser ED, Maulbecker-Armstrong C, Roitman M, et al. Are colon and rectal cancer two different tumor entities? A proposal to abandon the term colorectal cancer. Int J Mol Sci. 2018;19(9):2577. doi:10.3390/ijms19092577; 30200215 PMC6165083

[197] Kneis B, Wirtz S, Weber K, Denz A, Gittler M, Geppert C, et al. Colon cancer microbiome landscaping: differences in right- and left-sided colon cancer and a tumor microbiome-ileal microbiome association. Int J Mol Sci. 2023;24(4):3265. doi:10.3390/ijms24043265; 36834671 PMC9963782

[198] Baran B, Mert Ozupek N, Yerli Tetik N, Acar E, Bekcioglu O, Baskin Y. Difference between left-sided and right-sided colorectal cancer: a focused review of literature. Gastroenterol Res. 2018;11(4):264–73. doi:10.14740/gr1062w; 30116425 PMC6089587

[199] Tahara T, Yamamoto E, Suzuki H, Maruyama R, Chung W, Garriga J, et al. *Fusobacteriumin* colonic flora and molecular features of colorectal carcinoma. Cancer Res. 2014;74(5):1311–8. doi:10.1158/0008-5472.can-13-1865; 24385213 PMC4396185

[200] Mima K, Cao Y, Chan AT, Qian ZR, Nowak JA, Masugi Y, et al. *Fusobacterium nucleatum* in colorectal carcinoma tissue according to tumor location. Clin Transl Gastroenterol. 2016;7(11):e200. doi:10.1038/ctg.2016.53; 27811909 PMC5543402

[201] Yang Y, Weng W, Peng J, Hong L, Yang L, Toiyama Y, et al. *Fusobacterium nucleatum* increases proliferation of colorectal cancer cells and tumor development in mice by activating toll-like receptor 4 signaling to nuclear factor-κB, and up-regulating expression of microRNA-21. Gastroenterology. 2017;152(4):851–66.e24. doi:10.1053/j.gastro.2016.11.018; 27876571 PMC5555435

[202] Hardman RA, Afshari CA, Barrett JC. Involvement of mammalian MLH1 in the apoptotic response to peroxide-induced oxidative stress. Cancer Res. 2001;61(4):1392–7; 11245440

[203] Woerner SM, Kloor M, Von Knebel Doeberitz M, Gebert JF. Microsatellite instability in the development of DNA mismatch repair deficient tumors. Cancer Biomark. 2006;2(1–2):69–86. doi:10.3233/cbm-2006-21-208; 17192061

[204] Chen F, Arseven OK, Cryns VL. Proteolysis of the mismatch repair protein MLH1 by caspase-3 promotes DNA damage-induced apoptosis. J Biol Chem. 2004;279(26):27542–8. doi:10.1074/jbc.M400971200; 15087450

[205] Liang L, Kong C, Li J, Liu G, Wei J, Wang G, et al. Distinct microbes, metabolites, and the host genome define the multi-omics profiles in right-sided and left-sided colon cancer. Microbiome. 2024;12(1):274. doi:10.1186/s40168-024-01987-7; 39731152 PMC11681701

[206] Zhang C, Ma M, Zhao Z, Feng Z, Chu T, Wang Y, et al. Gut mucosal microbiota profiles linked to development of positional-specific human colorectal cancer. AIMS Microbiol. 2024;10(4):812–32. doi:10.3934/microbiol.2024035; 39628718 PMC11609426

[207] Hamada T, Nowak JA, Milner DA Jr, Song M, Ogino S. Integration of microbiology, molecular pathology, and epidemiology: a new paradigm to explore the pathogenesis of microbiome-driven neoplasms. J Pathol. 2019;247(5):615–28. doi:10.1002/path.5236; 30632609 PMC6509405

[208] Watson KM, Gardner IH, Anand S, Siemens KN, Sharpton TJ, Kasschau KD, et al. Colonic microbial abundances predict adenoma formers. Ann Surg. 2023;277(4):e817–24. doi:10.1097/sla.0000000000005261; 35129506 PMC9023594

[209] Liu J, Huang X, Chen C, Wang Z, Huang Z, Qin M, et al. Identification of colorectal cancer progression-associated intestinal microbiome and predictive signature construction. J Transl Med. 2023;21(1):373. doi:10.1186/s12967-023-04119-1; 37291572 PMC10249256

[210] Sun L, Zhu Z, Jia X, Ying X, Wang B, Wang P, et al. The difference of human gut microbiome in colorectal cancer with and without metastases. Front Oncol. 2022;12:982744. doi:10.3389/fonc.2022.982744; 36387258 PMC9665410

[211] Hirsch BR, Zafar SY. Capecitabine in the management of colorectal cancer. Cancer Manage Res. 2011;3:79–89. doi:10.2147/cmr.s11250; 21629830 PMC3097797

[212] Kong J, Zhang Z, Musch MW, Ning G, Sun J, Hart J, et al. Novel role of the vitamin D receptor in maintaining the integrity of the intestinal mucosal barrier. Am J Physiol Gastrointest Liver Physiol. 2008;294(1):1–30. doi:10.1152/ajpgi.00398.2007; 17962355

[213] Zhang YG, Wu S, Sun J. Vitamin D, vitamin D receptor and tissue barriers. Tissue Barriers. 2013;1(1):e23118. doi:10.4161/tisb.23118; 24358453 PMC3865708

[214] Sun J, Kong J, Duan Y, Szeto FL, Liao A, Madara JL, et al. Increased NF-κB activity in fibroblasts lacking the vitamin D receptor. Am J Physiol Endocrinol Metab. 2006;291(2):1–8. doi:10.1152/ajpendo.00590.2005; 16507601

[215] Adams JS, Hewison M. Unexpected actions of vitamin D: new perspectives on the regulation of innate and adaptive immunity. Nat Clin Pract Endocrinol Metab. 2008;4(2):80–90. doi:10.1038/ncpendmet0716; 18212810 PMC2678245

[216] Kamen DL, Tangpricha V. Vitamin D and molecular actions on the immune system: modulation of innate and autoimmunity. J Mol Med. 2010;88(5):441–50. doi:10.1007/s00109-010-0590-9; 20119827 PMC2861286

[217] Srichomchey P, Sukprasert S, Khulasittijinda N, Voravud N, Sahakitrungruang C, Lumjiaktase P. Vitamin D3 supplementation promotes regulatory T-cells to maintain immune homeostasis after surgery for early stages of colorectal cancer. In Vivo. 2023;37(1):286–93. doi:10.21873/invivo.13078; 36593062 PMC9843780

[218] Thomas RL, Jiang L, Adams JS, Xu ZZ, Shen J, Janssen S, et al. Vitamin D metabolites and the gut microbiome in older men. Nat Commun. 2020;11:5997. doi:10.1038/s41467-020-19793-8; 33244003 PMC7693238

[219] He Z, Xie H, Xu H, Wu J, Zeng W, He Q, et al. Chemotherapy-induced microbiota exacerbates the toxicity of chemotherapy through the suppression of interleukin-10 from macrophages. Gut Microbes. 2024;16(1):2319511. doi:10.1080/19490976.2024.2319511; 38400752 PMC10896127

