# Supplementary material for: The Role of the Gut Microbiome in Clinical Outcomes of Colorectal Cancer: A Systematic Review (2020–2025)
Source: Oncol Res. 2026 Feb 24;34(3):3. doi: 10.32604/or.2025.070281 (PMC12963652; doi:10.32604/or.2025.070281)
Supplement: Supplementary file 2 [file OncolRes-34-70281-s002.docx]

| **Section and Topic** | **Item No** | **Checklist item** | **Reported on Page Number/Line Number** | **Reported on Section/Paragraph** |
| --- | --- | --- | --- | --- |
| **TITLE** | | |  |  |
| Title | 1 | Identify the report as a systematic review. | Page 1 | Title |
| **ABSTRACT** | | |  |  |
| Abstract | 2 | See the PRISMA 2020 for Abstracts checklist. | Page 2 - 3 | Abstract |
| **INTRODUCTION** | | |  |  |
| Rationale | 3 | Describe the rationale for the review in the context of existing knowledge. | Page 4 | Introduction |
| Objectives | 4 | Provide an explicit statement of the objective(s) or question(s) the review addresses. | Page 4 | Introduction (Paragraph 5) |
| **METHODS** | | |  |  |
| Eligibility criteria | 5 | Specify the inclusion and exclusion criteria for the review and how studies were grouped for the syntheses. | Pages 4 - 7 | Materials and Methods (Literature Research Strategy, Selection Criteria, and Review Methods) |
| Information sources | 6 | Specify all databases, registers, websites, organisations, reference lists and other sources searched or consulted to identify studies. Specify the date when each source was last searched or consulted. | Page 4 | Materials and Methods (Literature Research Strategy, Paragraphs 3 and 4) |
| Search strategy | 7 | Present the full search strategies for all databases, registers and websites, including any filters and limits used. | Pages 4 - 7 | Materials and Methods (Literature Research Strategy, Table 1, Selection Criteria and Review Methods) |
| Selection process | 8 | Specify the methods used to decide whether a study met the inclusion criteria of the review, including how many reviewers screened each record and each report retrieved, whether they worked independently, and if applicable, details of automation tools used in the process. | Pages 4 - 9 | Materials and Methods (Literature Research Strategy, Selection Criteria and Review Methods, Figure 1, Quality Assessment Review) |
| Data collection process | 9 | Specify the methods used to collect data from reports, including how many reviewers collected data from each report, whether they worked independently, any processes for obtaining or confirming data from study investigators, and if applicable, details of automation tools used in the process. | Pages 4 - 7 | Materials and Methods (Literature Research Strategy, Selection Criteria, and Review Methods, Figure 1) |
| Data items | 10a | List and define all outcomes for which data were sought. Specify whether all results that were compatible with each outcome domain in each study were sought (e.g. for all measures, time points, analyses), and if not, the methods used to decide which results to collect. | Page 5 | Materials and Methods (Literature Research Strategy, Paragraph 2) |
|  | 10b | List and define all other variables for which data were sought (e.g. participant and intervention characteristics, funding sources). Describe any assumptions made about any missing or unclear information. | Pages 4 - 5, 9 - 11, and 15-17 | Materials and Methods (Literature Research Strategy, Paragraph 2) and Results (Table 2 & Table 3) |
| Study risk of bias assessment | 11 | Specify the methods used to assess risk of bias in the included studies, including details of the tool(s) used, how many reviewers assessed each study and whether they worked independently, and if applicable, details of automation tools used in the process. | Pages 7- 9 | Material and Methods (Quality Assessment Review, Figure 2 and Figure 3) |
| Effect measures | 12 | Specify for each outcome the effect measure(s) (e.g. risk ratio, mean difference) used in the synthesis or presentation of results. | Page 7 | Material and Methods (Selection Criteria and Review Methods, Paragraph 5 |
| Synthesis methods | 13a | Describe the processes used to decide which studies were eligible for each synthesis (e.g. tabulating the study intervention characteristics and comparing against the planned groups for each synthesis (item #5)). | Pages 4 - 7 | Materials and Methods (Literature Research Strategy, Selection Criteria and Review Methods) |
|  | 13b | Describe any methods required to prepare the data for presentation or synthesis, such as handling of missing summary statistics, or data conversions. | Pages 5 - 8 | Materials and Methods (Selection Criteria and Review Methods, Quality Assessment Review) |
|  | 13c | Describe any methods used to tabulate or visually display results of individual studies and syntheses. | Pages 7- 8, 8 – 11, and 15 - 18 | Materials and Methods (Quality Assessment Review, Figure 2 & 3) and Results (Table 2, 3 & 4) |
|  | 13d | Describe any methods used to synthesize results and provide a rationale for the choice(s). If meta-analysis was performed, describe the model(s), method(s) to identify the presence and extent of statistical heterogeneity, and software package(s) used. | Page 7 | Material and Methods (Selection Criteria and Review Methods, Paragraph 5 |
|  | 13e | Describe any methods used to explore possible causes of heterogeneity among study results (e.g. subgroup analysis, meta-regression). | Pages 7 and 24 - 30 | Material and Methods (Selection Criteria and Review Methods, Paragraph 5) and Discussion (“Differences across Microbiome Compositions and Functional Redundancy” to “Therapeutic Intervention and Microbiome”) |
|  | 13f | Describe any sensitivity analyses conducted to assess robustness of the synthesized results. | NA | NA |
| Reporting bias assessment | 14 | Describe any methods used to assess risk of bias due to missing results in a synthesis (arising from reporting biases). | Page 7 - 8 | Material and Methods (Quality Assessment Review) |
| Certainty assessment | 15 | Describe any methods used to assess certainty (or confidence) in the body of evidence for an outcome. | Pages 8 and 17-18 | Material and Methods (Quality Assessment Review, Paragraph 5) and Results (Table 4) |
| **RESULTS** | | |  |  |
| Study selection | 16a | Describe the results of the search and selection process, from the number of records identified in the search to the number of studies included in the review, ideally using a flow diagram. | Page 6 | Material and Methods (Selection Criteria and Review Methods, Figure 1) |
|  | 16b | Cite studies that might appear to meet the inclusion criteria, but which were excluded, and explain why they were excluded. | Page 6 | Material and Methods (Selection Criteria and Review Methods, Figure 1) |
| Study characteristics | 17 | Cite each included study and present its characteristics. | Pages 8 - 20 | Results |
| Risk of bias in studies | 18 | Present assessments of risk of bias for each included study. | Pages 7 - 8 | Materials and Methods (Quality Assessment Review, Figures 2 & 3) |
| Results of individual studies | 19 | For all outcomes, present, for each study: (a) summary statistics for each group (where appropriate) and (b) an effect estimate and its precision (e.g. confidence/credible interval), ideally using structured tables or plots. | Pages 8 - 20 | Results |
| Results of syntheses | 20a | For each synthesis, briefly summarise the characteristics and risk of bias among contributing studies. | Pages 7 – 20 | Materials and Methods (Quality Assessment Review, Figures 2 & 3) and Results |
|  | 20b | Present results of all statistical syntheses conducted. If meta-analysis was done, present for each the summary estimate and its precision (e.g. confidence/credible interval) and measures of statistical heterogeneity. If comparing groups, describe the direction of the effect. | Pages 8 - 20 | Results |
|  | 20c | Present results of all investigations of possible causes of heterogeneity among study results. | Pages 17 – 20, and 24 - 30 | Results (Table 4) and Discussion (“Differences across Microbiome Compositions and Functional Redundancy” to “Therapeutic Intervention and Microbiome”) |
|  | 20d | Present results of all sensitivity analyses conducted to assess the robustness of the synthesized results. | NA | NA |
| Reporting biases | 21 | Present assessments of risk of bias due to missing results (arising from reporting biases) for each synthesis assessed. | Pages 7 - 8 | Materials and Methods (Quality Assessment Review, Figures 2 & 3) |
| Certainty of evidence | 22 | Present assessments of certainty (or confidence) in the body of evidence for each outcome assessed. | Pages 19 -20 | Results (Table 4) |
| **DISCUSSION** | | |  |  |
| Discussion | 23a | Provide a general interpretation of the results in the context of other evidence. | Pages 18 – 30 | Discussion |
|  | 23b | Discuss any limitations of the evidence included in the review. | Pages 20 - 30 | Discussion (“Differences across Microbiome Compositions and Functional Redundancy” to “Therapeutic Intervention and Microbiome”) |
|  | 23c | Discuss any limitations of the review processes used. | Page 31 | Discussion (Limitations of this Review) |
|  | 23d | Discuss implications of the results for practice, policy, and future research. | Pages 24 – 30 | Discussion (“Differences across Microbiome Compositions and Functional Redundancy” to “Therapeutic Intervention and Microbiome”) |
| **OTHER INFORMATION** | | |  |  |
| Registration and protocol | 24a | Provide registration information for the review, including register name and registration number, or state that the review was not registered. | Page 4 | Material and Methods (Literature Research Strategy, Paragraph 1) |
|  | 24b | Indicate where the review protocol can be accessed, or state that a protocol was not prepared. | Page 4 | Material and Methods (Literature Research Strategy, Paragraph 1) |
|  | 24c | Describe and explain any amendments to information provided at registration or in the protocol. | Page 4 | Material and Methods (Literature Research Strategy, Paragraph 1) |
| Support | 25 | Describe sources of financial or non-financial support for the review, and the role of the funders or sponsors in the review. | Page 32 | Funding Statement |
| Competing interests | 26 | Declare any competing interests of review authors. | Page 32 | Conflicts of Interest |
| Availability of data, code and other materials | 27 | Report which of the following are publicly available and where they can be found: template data collection forms; data extracted from included studies; data used for all analyses; analytic code; any other materials used in the review. | Page 32 | Availability of Data and Materials |

*As the checklist was provided upon initial submission, the page number/line number reported may be changed due to copyediting and may not be referable in the published version. In this case, the section/paragraph may be used as an alternative reference.
